# *De novo* formation of early endosomes during Rab5-to-Rab7a transition

**DOI:** 10.1242/jcs.254185

**Published:** 2021-04-27

**Authors:** Frode Miltzow Skjeldal, Linda Hofstad Haugen, Duarte Mateus, Dominik M. Frei, Anna Vik Rødseth, Xian Hu, Oddmund Bakke

**Affiliations:** Department of Biosciences, University of Oslo, 0371 Oslo, Norway

**Keywords:** Endosomal biogenesis, Endosome maturation, FRAP, Rab5, Rab7a

## Abstract

Rab5 and Rab7a are the main determinants of early and late endosomes and are important regulators of endosomal progression. The transport from early endosomes to late endosome seems to be regulated through an endosomal maturation switch, where Rab5 is gradually exchanged by Rab7a on the same endosome. Here, we provide new insight into the mechanism of endosomal maturation, for which we have discovered a stepwise Rab5 detachment, sequentially regulated by Rab7a. The initial detachment of Rab5 is Rab7a independent and demonstrates a diffusion-like first-phase exchange between the cytosol and the endosomal membrane, and a second phase, in which Rab5 converges into specific domains that detach as a Rab5 indigenous endosome. Consequently, we show that early endosomal maturation regulated through the Rab5-to-Rab7a switch induces the formation of new fully functional Rab5-positive early endosomes. Progression through stepwise early endosomal maturation regulates the direction of transport and, concomitantly, the homeostasis of early endosomes.

## Abstract (precis)

**Highlighted Article:** A crucial step in endosomal maturation is the exchange of Rab5 with Rab7a, and we show that this two-phase exchange is finalized by the formation of Rab5-positive early endosomes.

## INTRODUCTION

Endosomal trafficking is carefully regulated through the mechanisms of endosomal progression. In general, two models have been characterized to explain the trafficking through the endocytic pathway (Helenius et al., 1983); i.e. the endosomal shuttling model (Griffiths and Gruenberg, 1991) and the maturation model (Dunn and Maxfield, 1992; Murphy, 1991). The shuttling model is based on preexisting early endosomes that function as a stationary recycling compartment. From this compartment, carrier vesicles are released to recycle back to the plasma membrane or to interact and fuse with late endosomes (Griffiths and Gruenberg, 1991). The maturation model emphasizes the function of the early endosome as a transient compartment, where transport between early to late endosomes is driven by endosomal maturation. With this model, the early endosomes are constantly regenerated through endosomal maturation and are not stationary compartments (Murphy, 1991). Later shuttling models have also shown that the carrier vesicles, released by the sorting early endosome, go through maturation, and traffic between the early and late endosomes (Scott et al., 2014). The early endosomal maturation process involves changes in the membrane markers as well as the lipid composition of the endosomes (Ebner et al., 2019; Huotari and Helenius, 2011). This pivotal moment in the endocytic pathway is crucial for the sorting of internalized macromolecules and receptors, and it is regulated through exchange of the two GTPases Rab5 and Rab7a in the endocytic pathway (Rink et al., 2005). Early endosomes are characterized by the presence of the small GTPase Rab5 on the membrane, responsible for the recruitment of several effectors that determine the identity and functionality of this compartment (Behnia and Munro, 2005; Huotari and Helenius, 2011). The recruitment of Rab5 to the early endosome membrane occurs after a transition from cytosolic inactive Rab5-GDP to its active GTP-bound form, promoted by the Rab5 effector Rabex5 (officially known as RABGEF1) (Barr and Lambright, 2010). Once on the endosome, Rab5 further stimulates its own recruitment through a positive feedback loop (Lippe et al., 2001). The early endosome will then change characteristics, becoming increasingly acidic, and initiates the formation of intraluminal vesicles, thereby, retaining cargo destined for lysosomal degradation (Scott et al., 2014). One crucial step in this progression is the conversion from early to late endosome through maturation, where the late endosome is formed by an exchange of GTPases, i.e. Rab5 for Rab7a (Rink et al., 2005). The replacement of Rab5 with Rab7a is orchestrated by Mon1 (also known as SAND-1) and Ccz1, as these two cytosolic factors bind Rab5-GTP on the membrane of the early endosome. They initiate recruitment of Rab7a, while promoting the dissociation of Rabex5 from the endosome, thereby, leading to detachment of Rab5 (Poteryaev et al., 2010). Specifically, the complex between Mon1 and Ccz1 is a Rab5 effector, i.e. directly promoting recruitment of Rab7a to the maturing endosome, showing guanine nucleotide exchange factor (GEF) activity of Mon1–Ccz1 towards Rab7a (Langemeyer et al., 2020; Nordmann et al., 2010). Once this step of maturation is completed, the endosome becomes a Rab7a-positive late endosome that will mature and initiate lysosomal degradation (Hesketh et al., 2018; Vanlandingham and Ceresa, 2009).

Endosomal enlargement is achieved by expression of the specialized major histocompatibility complex (MHC) class II chaperone invariant chain (Ii) (Nordeng et al., 2002; Stang and Bakke, 1997). We, therefore, analyzed endosomes in HeLa cells and enlarged endosomes in MDCK-Ii cells, i.e. MDCK cells that express the Ii chain. The cytosolic tail of Ii has fusogenic properties that can induce the enlargement of endosomes and prolong the lifetime of an early endosome (Engering et al., 1998; Nordeng et al., 2002; Romagnoli et al., 1993; Stang and Bakke, 1997) without inhibiting the transition from early to late endosome (Engering et al., 1998; Nordeng et al., 2002; Romagnoli et al., 1993; Stang and Bakke, 1997). This effect of Ii has been previously utilized to increase and study endosomal fusion/fission, coat binding kinetics, morphology and maturation (Bergeland et al., 2008; Haugen et al., 2017; Landsverk et al., 2011; Margiotta et al., 2020; Skjeldal et al., 2012).

The Rab GTPase cycle has become the standard when defining endosomal progression and maturation (Rink et al., 2005). However, by performing live cell imaging, we were able to provide new data on endosomal maturation and progression by investigating the Rab5-to-Rab7a exchange. Rab5 detachment from the maturing endosome occurred in a stepwise manner, with the initial detachment being independent of Rab7a. Completion of the Rab5 detachment, however, depended on endosomal Rab7a recruitment. During completion of the detachment phase, Rab5 assembled into a ‘hot spot’ on the endosomal membrane and this concentrated area of Rab5 can act as a physical cue for the new formation of Rab5-positive endosomes.

This previously undocumented formation of a nascent Rab5-positive endosome from a ‘mother’ endosome in transition might explain how homeostasis of the endocytic pool is preserved through the biogenesis of early endosomes during the Rab5-to-Rab7a exchange.

## RESULTS

### Detachment of Rab5 occurs in two phases

**Fig. 1.**
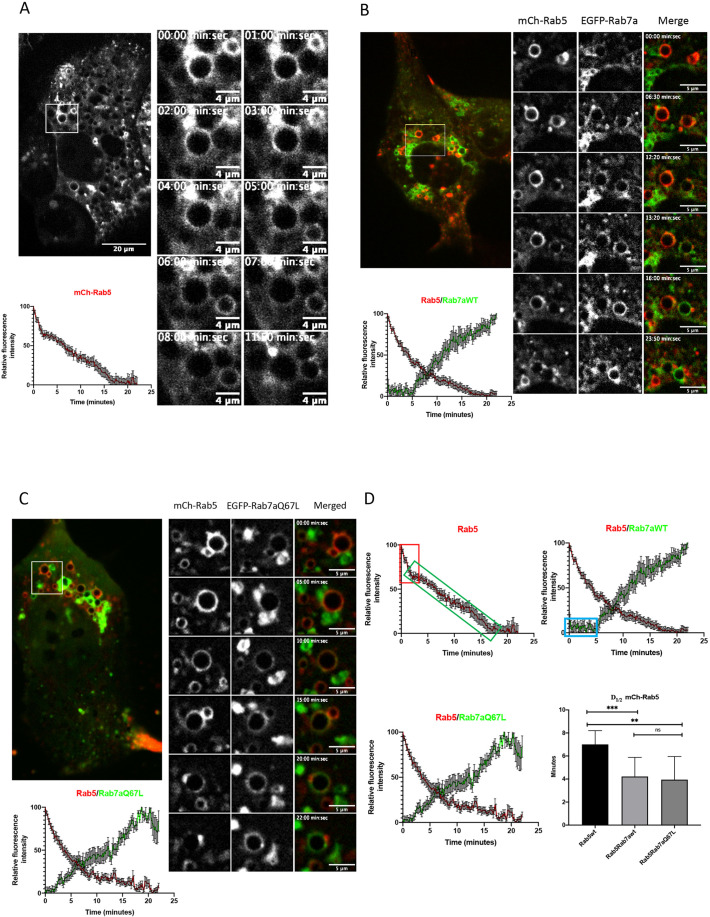
**The Rab5 detachment occurs in two phases.** (A) MDCK cells were stably transfected with invariant chain (MDCK-Ii cells) under the control of an inducible metallothionein promoter. The cells were treated with 25 µM CdCl_2_ overnight to induce endosomal enlargement and co-transfected with mCh-Rab5. The graph shows the average of mCh-Rab5 detachment on 14 single mCh-Rab5 positive endosomes as fluorescence intensity over time. The figures to the right shows time series of a representative example of mCh-Rab5 detachment from one endosome (white box). (B) MDCK-Ii cells were co-transfected with mCh-Rab5 and EGFP-Rab7a. The graph shows the average of mCh-Rab5 detachment and EGFP-Rab7a recruitment for 14 single mCh-Rab5 positive endosomes as fluorescence intensity over time. White box signifies a representative example of mCh-Rab5 detachment and EGFP-Rab7a recruitment on one endosome (time series in the right panel). (C) MDCK-Ii cells were co-transfected with mCh-Rab5 and EGFP-Rab7aQ67L. The graph shows the average of mCh-Rab5 detachment and EGFP-Rab7aQ67L recruitment for 14 single mCh-Rab5 positive endosomes as fluorescence intensity over time. White box signifies a representative example of mCh-Rab5 detachment and EGFP-Rab7aQ67L recruitment on one endosome (time series in the right panel). (D) This figure (based on A-C) illustrates the detachment pattern of mCh-Rab5 and the recruitment pattern of EGFP-Rab7a/EGFP-Rab7aQ67L as fluorescence intensity over time. Red box (upper left graph) shows the initial rapid detachment phase of single transfected mCh-Rab5, followed by a slower convergence phase (green box). Upper right graph shows a lag phase (blue box) of EGFP-Rab7a recruitment in cells double transfected with mCh-Rab5 and EGFP-Rab7a, whereas the lower left graph shows the lack of the lag phase in cells double transfected with mCh-Rab5 and EGFP-Rab7aQ67L. The bar graph (bottom right) shows the calculated mCh-Rab5 detachment halftime (D1/2) in single- and double-transfected cells. D1/2 was quantified by nonlinear regression (Prism) and averaged over 14 measurements. In single-transfected mCh-Rab5 cells, from A, D1/2=7.005 min±1.195 min s.d., N=14. Double-transfected cells, mCh-Rab5 and EGFP-Rab7a, from B, D1/2=4.216 min±1.659 min s.d., N=14. Double-transfected cells, mCh-Rab5 and EGFP-Rab7aQ67L, from C, D1/2 = 3.865 min±1.958 min s.d., N=14. The analysis shows a significant difference between mCh-Rab5 D1/2 in the control experiment compared to double transfected with EGFP-Rab7a (*P*<0.0007), or EGFP-Rab7aQ67L (*P*<0.0012), whereas mCh-Rab5 D1/2 between the double-transected experiments were not significant (ns). Significance was determined using a paired two-tailed *t*-test. **P=0.0012, ***P=0.0007. Data are mean±s.d.

Endosomal progression, from early to late endosomes, is carefully regulated by the transition from a Rab5-positive to a Rab7a-positive endosome. To better understand this pivotal transition, we performed a live cell study of this particular conversion on endosomes of MDCK-Ii cells and HeLa cells (Nordeng et al., 2002; Stang and Bakke, 1997). MDCK-Ii cells were transfected with mCh-Rab5 alone (control), or co-transfected with mCh-Rab5 and EGFP-tagged Rab7a wild type (wt) or with mCh-Rab5 and the EGFP-tagged constitutively active mutant Rab7aQ67L (Fig. 1A–C). During early endosomal progression, we observed numerous homotypic fusion events (Skjeldal et al., 2012) in which the endosomes increased in size, preparing for the transition from early to late endosome. However, before the transition of Rab5-positive to Rab7a-positive endosomes, early endosomal homotypic fusion events ceased and we initiated the endosomal analysis of the mCh-Rab5 coat detachment and concomitant EGFP-Rab7a acquisition on individual endosomes (Fig. 1A–C).

In control cells (MDCK-Ii cells transfected with mCh-Rab5), we followed single mCh-Rab5-positive endosomes and plotted the detachment of mCh-Rab5 over time (Fig. 1A, Movie 1). Similarly to previously observed Rab5-to-Rab7a conversion (Rink et al., 2005), Rab5 gradually detached from the maturing endosome and the coat became mCh-Rab5 negative (Fig. 1A). To better understand the mCh-Rab5 coat dynamics during maturation we measured the total time of detachment and calculated the coat detachment halftime (D_1/2_; see Materials and Methods). In the control cells (Fig. 1A), we measured D_1/2_ for mCh-Rab5 to be 7.0±1.2 min (Fig. 1D, graph). To further analyze whether higher expression levels of Rab7a affect the endosomal detachment of Rab5, we co-transfected MDCK-Ii cells with mCh-Rab5 and EGFP-Rab7a wt (Fig. 1B, Movie 2). As has been previously shown (Rink et al., 2005), we also observed a gradual exchange of Rab5 with Rab7a (Fig. 1). Analysis further showed that increased Rab7a expression induced a decrease in D_1/2_ for mCh-Rab5, 4.2±1.7 min (Fig. 1B, graph). We measured a similar effect on the D_1/2_ of mCh-Rab5, when we expressed the GTP-bound form of Rab7a, EGFP-Rab7aQ67L. In that case the mCh-Rab5 D_1/2_ was 3.9±1.9 min (Fig. 1C, graph, Movie 3), indicating that Rab7a – when recruited to the endosomal membrane – regulates Rab5 detachment during endosomal conversion. When comparing the D_1/2_ values between the three experiments, we found a significant difference in the D_1/2_ for mCh-Rab5 when the cells were co-transfected with Rab7a or Rab7aQ67L (Fig. 1D).

Furthermore, the characteristics of the detachment graph in the control experiment revealed a new pattern of endosomal coat dynamics. We could clearly classify two different phases that lead to mCh-Rab5 detachment; (1) the rapid initial (2–3 min) detachment phase (Fig. 1D, red box), followed by (2) the slower completion phase (Fig. 1D, green box). In another set of experiments, cells were co-transfected with mCh-Rab5 and EGFP-Rab7a wt. Here, we measured a delay of ∼5 min, i.e. a lag period, before Rab7a was recruited to the endosome (Fig. 1D, blue box), coinciding with the Rab5 initial detachment. In fact, ∼50% of Rab5 detached before any Rab7a was detected on the endosomal membranes. However, when cells were expressing the GTP-bound Rab7a mutant EGFP-Rab7aQ67L, we did not observe any pronounced lag period.

These experiments showed us that the mCh-Rab5 detachment dynamics can be divided in two phases, a brief initial phase and a longer completion phase. In addition, these experiments indicate that recruitment of Rab7a to the maturing endosome occurs only after initial detachment of Rab5 and that Rab7a is important for completion phase – highlighted by the finding that Rab7a expression led to a decrease of D_1/2_ for Rab5.

### Initial Rab5 detachment is Rab7a independent

**Fig. 2.**
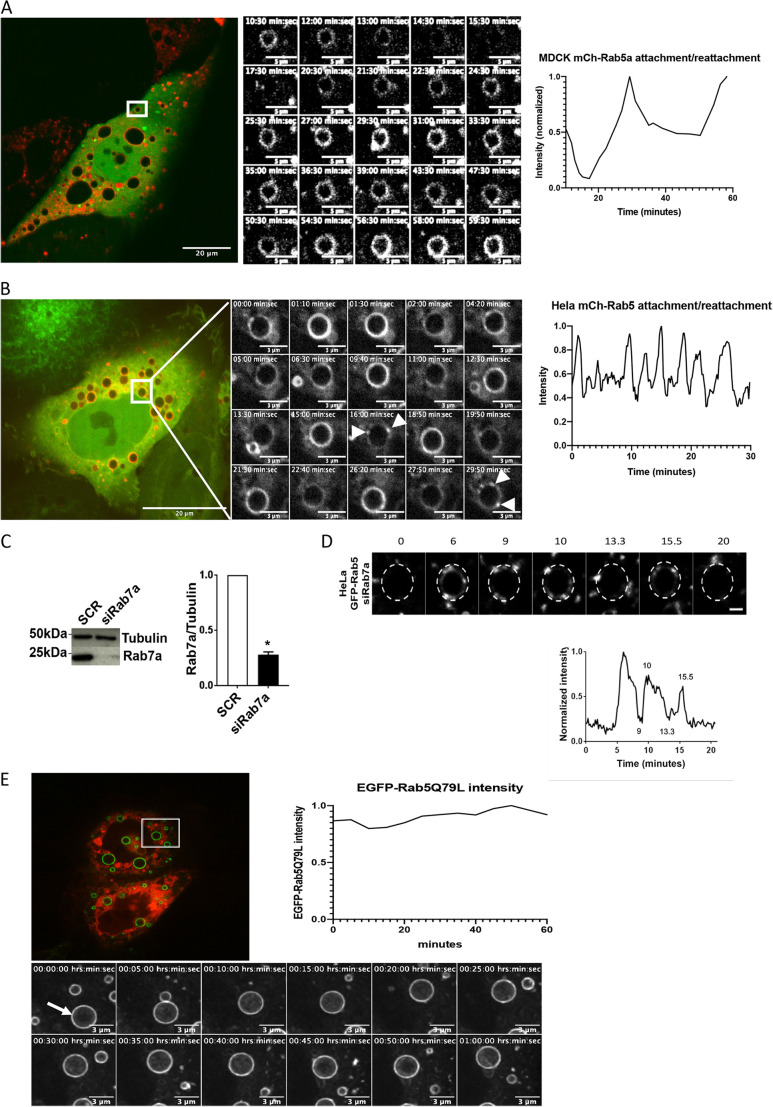
**Initial detachment of Rab5 is independent of Rab7a.** (A) MDCK expressing Ii induced enlarged endosomes co-transfected with mCh-Rab5 and the dominant-negative mutant EGFP-Rab7aT22N. The boxed area in the panel on the right is shown as a series of images that show detachment and reattachment of mCh-Rab5 induced by the dominant-negative mutant (middle panel). Representative mCh-Rab5 detachment/reattachment over time is shown on the right. (B) HeLa cells transfected with mCh-Rab5 and EGFP-Rab7aT22N show detachment and reattachment with higher frequency than in a similar experiment in MDCK cells. The boxed area in the panel on the right is shown as an image series over 30 min; arrowheads indicate transient domains. Representative mCh-Rab5 detachment/reattachment over time is plotted on the right. (C) Whole-cell lysate from SCR (scrambled/control) and siRab7a in HeLa cells were separated by SDS-PAGE and immunoblotted for the indicated proteins. The intensities of the bands were quantified using densiometric analysis and plotted relative to the SCR as mean±s.e.m., *n*=3. (D) Image series of an EGFP-Rab5-positive endosome in HeLa cells over 20 min, showing typical detachment and reattachment induced by knockdown of Rab7a, Scale bar: 1 µm. The normalized intensity of the endosome depicted in the montage plotted over time is shown underneath. (E) EGFP-Rab5Q79L-positive endosomes (green) in HeLa cell transfected with dominant-negative DsRed-Rab7aT22N (whole-cell image on left). Below: image series (over 1 h): endosomes in these cells did not show the typical detachment/reattachment pattern of Rab5. Fluorescence intensity of EGFP-Rab5Q79L on the representative endosome (white arrow) is plotted on the right.

We find that the endosomal maturation starts with an initial detachment of Rab5 before any Rab7a is detected on the membrane (Fig. 1). Our analysis showed that the onset of Rab7a attachment to the endosomes is occurring after Rab5 has started to detach (Fig. 1D, red and blue box) at a time where >50% of Rab5 has left the endosome. To further address the significance of Rab7a recruitment for the dissociation of mCh-Rab5 from the endosome, we co-transfected mCh-Rab5 with the dominant-negative mutant of Rab7a (Rab7aT22N) tagged with EGFP (EGFP-Rab7aT22N) or depleted Rab7a by using small interfering RNA targeting Rab7a (siRab7a) in HeLa and MDCK-Ii cells (Fig. 2). Rab7aT22N is locked in the GDP state and, thus, located in the cytosol, acts as a dominant-negative mutant of Rab7a (Bucci et al., 2000; Feng et al., 1995).

In MDCK-Ii cells co-transfected with mCh-Rab5 and EGFP-Rab7aT22N (Fig. 2A), we observed that EGFPRab7aT22N caused the endosomes to remain mCh-Rab5 positive for longer – in fact, mCh-Rab5 detached and reattached but never completed the detachment (Fig. 2A). Performing the same experiment in HeLa cells we observed an increase in size of the mCh-Rab5-positive endosomes in EGFP-Rab7aT22N-transfected cells (Fig. 2B, Movie 4). Single endosomal analysis showed a similar and repetitive cyclic pattern, i.e. detachment and reattachment of mCh-Rab5, but with a higher frequency (Fig. 2B). The frequency seemed to be quite stable and the cycle period for different endosomes varied from 2 min to 4 min (Fig. 2B). Furthermore, depletion of Rab7a by using RNAi in HeLa cells showed similar partial mCh-Rab5 detachment and reattachment (Fig. 2C,D). As shown in the graphs, the dissociation of mCh-Rab5 was fast and seemed to be similar to the first phase of mCh-Rab5 detachment (Fig. 1D, red box). Careful analysis of the endosomal membrane showed mCh-Rab5-positive domain as an intermediate state between maximum intensities during the intensity oscillations (Fig. 2B, white arrows). This indicates that the mCh-Rab5-positive endosomes failed to reach the second, i.e. completion phase, as Rab7a is inactivated or depleted and not recruited to endosomes in transition.

To search for a role of the Rab5 GDP/GTP cycle in this process, we co-transfected HeLa cells with constitutively active Rab5 mutant EGFP-Rab5Q79L that is unable to hydrolyze GTP and remains membrane-bound (Stenmark et al., 1994) together with DsRed-tagged Rab7aT22N (DsRed-Rab7aT22N). HeLa cells expressing EGFP-Rab5Q79L induced enlarged endosomal structures (Wegner et al., 2010) with a uniform EGFP-Rab5Q79L-positive membrane for more than 1 h (Fig. 2E). Under this condition, no cyclic variation was seen for EGFP-Rab5Q79L-positive membrane intensity (Fig. 2E). As the dominant-negative EGFP-Rab7aT22N did not induce detachment/reattachment of EGFP-Rab5Q79L, the initial detachment phase of Rab5 seems to be regulated by the rapid GDP/GTP cycle.

The above results demonstrate that Rab5 has the ability to transiently leave the maturing endosome independently of membrane-associated Rab7a, whereas Rab7a has to be recruited to the endosome membrane for Rab5 to complete its detachment. Overall, these results lead to a model where mCh-Rab5 detachment can be divided in two phases: (1) the initial phase that is Rab7a independent, and (2) the completion phase that is Rab7a dependent.

### Rab5-positive domains converge and leave the maturing endosome

**Fig. 3.**
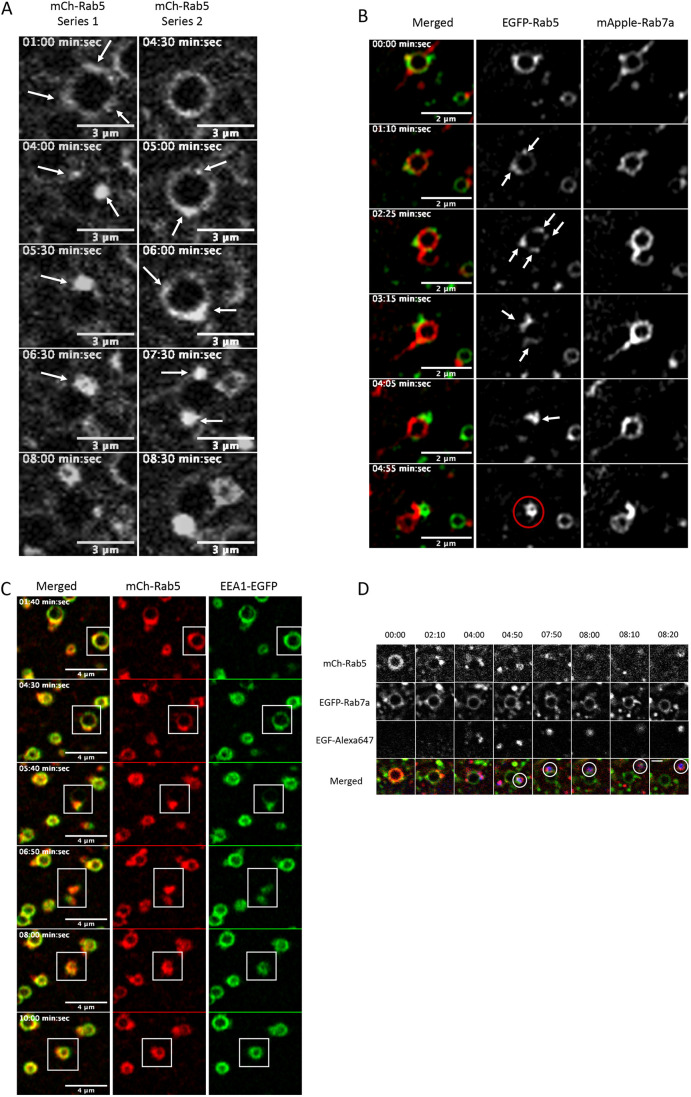
**Second phase of endosomal Rab5-to-Rab7a** **transition, Rab5 convergence.** (A) Two mCh-Rab5-positive endosomes in MDCK-Ii cells (Series 1 and 2) shown to go through the convergence phase. Arrows indicate the formation of mCh-Rab5-positive domains that converge to one or two main domains to accomplish mCh-Rab5 detachment. (B) HeLa cells expressing endosomes positive for EGFP-Rab5 and mApple-Rab7a (∼1 µm in diameter) going through the convergence phase of Rab5-to-Rab7a transition. Arrows indicate the formation of EGFP-Rab5-positive domains converging to one major domain to accomplish the Rab5 detachment through the formation of a (daughter) EGFP-Rab5-positive endosome (red circle). (C) HeLa cells expressing mCh-Rab5- and EEA1-EGFP-positive endosomes progressing through convergence phase to give rise to a newly formed mCh-Rab5- and EEA1-EGFP-positive endosome (white boxes indicate maturing endosome). (D) HeLa cells expressing mCh-Rab5- and EGFP-Rab7a-positive endosomes during uptake of EGF-Alexa-Fluor-647. The newly formed mCh-Rab5- and EGFP-Rab7a-positive endosome from the maturing endosome acquired EGF-Alexa-Fluor-647, showing a fully functional newly formed endosome (white circles). Scale bar: 2 µm.

During the analysis of single maturation events on Ii-enlarged endosomes in MDCK-Ii cells (Fig. 1) transfected with mCh-Rab5, we consistently observed the formation of endosomal Rab5-positive domains (Fig. 1, Fig. 3A, arrows). In a typical experiment presented in Fig. 3A, we followed mCh-Rab5-positive enlarged endosomes that changed from having a uniformly mCh-Rab5-positive membrane to acquiring several mCh-Rab5-positive domains. During Rab transition, these areas converge towards one (Fig. 3A, Series 1) or two (Fig. 3A, Series 2) distinct mCh-Rab5 domains to complete the second phase of maturation, the completion phase. Converging mCh-Rab5-positive domains appeared in all measured endosomes during the Rab5 completion phase. Similar experiments were performed using super-resolution imaging (Zeiss AiryScan) on HeLa cells transfected with EGFP-Rab5 and mApple-Rab7a. Here, the converging domains were confirmed on endosomes not enlarged with Ii (Fig. 3B, arrows, Movie 5). Similar to the previously measured enlarged endosomes, formation of EGFP-Rab5-positive domains on smaller endosomes started after the initial rapid decrease of Rab5 and persisted throughout the entire completion phase. Formation of endosomal microdomains and convergence to one or two major domains occurred in both Ii-enlarged MDCK-Ii cell endosomes and HeLa cell endosomes.

By following the fate of the converging endosomal Rab5 domains, we found that these domains, invariably, gave rise to newly formed mCh-Rab5- or EGFP-Rab5-positive endosomes (Fig. 3B, red circle). We found that >90% of the endosomes measured in Fig. 1A–C, matured by means of converging domains. Rab5-positive and converged domains leaving the maturing endosome seemed to be a prerequisite for the formation of new Rab5-positive early endosomes. We then asked whether this mechanism of maturation is unique for Rab5. Therefore, we, co-transfected cells with the well-known tethering protein early endosome antigen 1 (EEA1) tagged to GFP (EEA1-GFP) (Bergeland et al., 2008; Murray et al., 2016; Simonsen et al., 1998) and mCh-Rab5, and analyzed the coat detachment of the respective membrane-associated proteins. Detachment of the two membrane-associated proteins occurred simultaneously, and convergence towards a local endosomal hotspot was observed for both EEA1-GFP and mCh-Rab5. This indicates that both EEA1 and Rab5 follow a similar endosomal detachment pattern (Fig. 3C, white box, Movie 6). Further analysis of the Rab5-EEA1-positive vesicles showed that, after their release from the matured endosome, they instantly engaged in early homotypic fusion (Fig. 3C). EEA1 and Rab5 are kept on the membrane as a fully functional fusion machinery to ensure the newly released endosomes are instantly fully functional and engage in homotypic fusion. Endosomal maturation through convergence may work as a generic mechanism for several early endosomal-associated proteins. To further understand the maturation through convergence, we carried out similar experiments, transfecting HeLa or MDCK-Ii cells with EGFP-Rab22 and mCh-Rab5 (Fig. S1A,B, Fig. S2). Rab22 recruits Rabex5 to early endosomes for Rab5 activation (Zhu et al., 2009) and interacts with EEA1 (Kauppi et al., 2002). In cells transfected with EGFP-Rab22 and mCh-Rab5, we found the respective proteins to colocalize to endosomal structures (Fig. S1A,B, Fig. S2). Following the EGFP-Rab22 and mCh-Rab5-positive endosomes over time, we observed similar maturation through convergence that lead to a newly formed EGFP-Rab22- and mCh-Rab5-positive endosome (Figs S1, S2, white rings; Movies 7, 8, 9). EGFP-Rab22 was also transfected into HeLa cells together with mApple-Rab7a to analyze the transition from an EGFP-Rab22-positive endosome to one that is mApple-Rab7a positive (Fig. S3). Similarly to mCh-Rab5, we were able to detect a gradual transition from EGFP-Rab22-positive endosome to a mApple-Rab7a-positive endosome, and observed EGFP-Rab22 converging into a newly formed endosome (Fig. S3, white ring; Movie 10). Experiments were repeated by using EGFP-Rab4A in HeLa cells and MDCK-Ii cells co-transfected with mRFP-Hrs and EGFP-Eps15 (Haugen et al., 2017). These experiments confirmed the convergence maturation as previously observed (Figs S4, S5, white rings; Movies 11, 12).

Early endosomes work as sorting stations for protein destined for degradation or recycling (Huotari and Helenius, 2011). To better understand the function of newly formed mCh-Rab5 endosomes, we added EGF-Alexa-Fluor-647 to the cells. We found that the newly formed mCh-Rab5-positive endosome was able to recruit EGF-Alexa-Fluor-647-containing vesicles, indicating that it is primed for another round of sorting of EGF and/or EGFR (Fig. 3D).

### Rab7a recruitment during endosomal Rab5-to-Rab7a transition

**Fig. 4.**
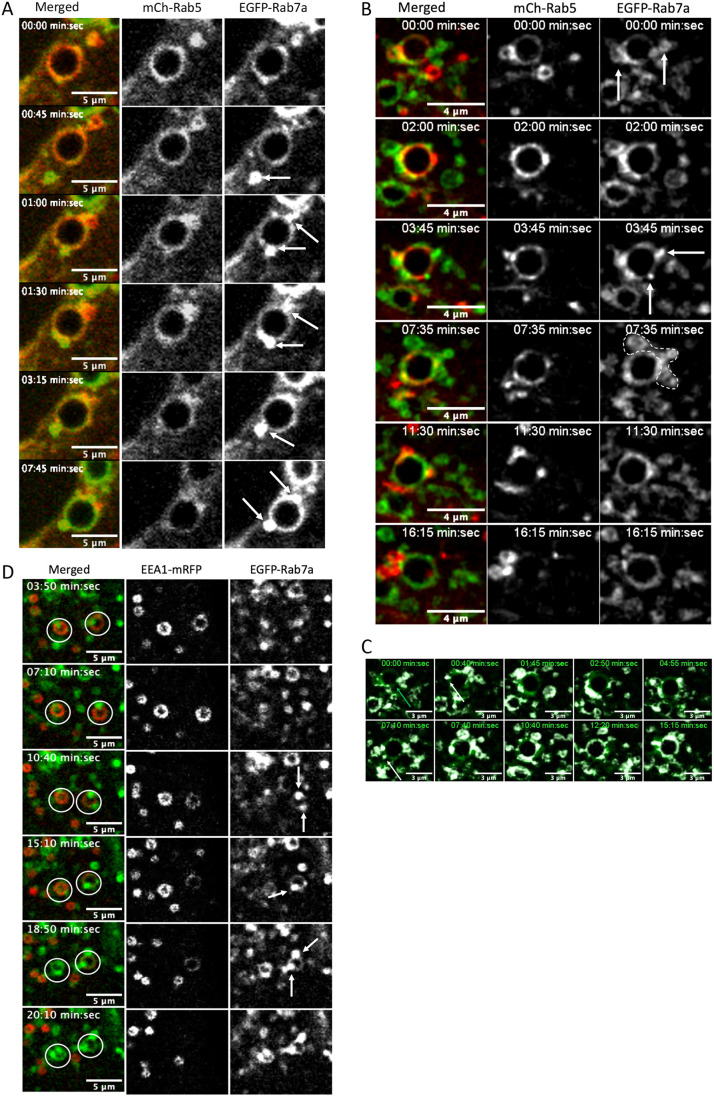
**Rab7a recruitment during endosomal Rab5-to-Rab7a** **transition.** (A) MDCK-Ii cells transfected with mCh-Rab5 and EGFP-Rab7a showing the gradual contribution of incoming EGFP-Rab7a throughout Rab5-to-Rab7a transition. During mCh-Rab5 detachment, EGFP-Rab7a-positive endosomes are recruited to the maturing endosome to provide the necessary EGFP-Rab7a. (B) HeLa cell expressing mCh-Rab5 and EGFP-Rab7a showing the gradual transition from Rab5- to Rab7a-positive endosomes. White arrows indicate incoming EGFP-Rab7a-positive vesicles recruited to the maturing endosome. The image sequence is a maximum projection of three *z*-planes with 0.32 µm between the confocal planes. (C) Image series showing EGFP-Rab7a minus the overlapping signal from mCh-Rab5 (white; see also Fig. 4B). The original EGFP-Rab7a is shown in green. The white signal represents the contribution of the incoming/interacting EGFP-Rab7a-positive vesicles recruited to the endosomal membrane during maturation. (D) MDCK-Ii cells transfected with EEA1-mRFP and EGFP-Rab7a showing recruitment of EGFP-Rab7a similar to that of the maturing endosome.

During the formation of mCh-Rab5-positive domains, we observed an increase of EGFP-Rab7a on the maturing endosome in domain-like structures (Fig. 1). Maturing endosomes in transfected HeLa and MDCK-Ii cells showed an increased number of interacting EGFP-Rab7a-positive vesicles during the mCh-Rab5 detachment (Fig. 4A,B, arrows and encircled area; Movies 13, 14). To better visualize these interacting EGFP-Rab7a-positive endosomes, we subtracted the overlapping signal between EGFP-Rab7a and mCh-Rab5. In Fig. 4D, the subtracted signal is shown in white and the original EGFP-Rab7a is shown in green (merged image). By using this method, we can track the contribution from the interacting EGFP-Rab7a-positive vesicles to the maturing endosome (Fig. 4C; Movie 15). The recruitment of EGFP-Rab7a to the maturing endosome seems to be divided in two phases, where recruitment in the first phase (Fig. 4C, green arrow) may be recruited from cytosol and that of the second phase through vesicular interaction at EGFP-Rab7a-positive microdomains (Fig. 4A–C, white arrows). This experiment was repeated with MDCK-Ii cells co-transfected with EEA1-mRFP and EGFP-Rab7a, and which showed the same result (Fig. 4D, Movie 16), i.e. endosomes in transition seem to acquire Rab7a through a fusion-like or ‘kiss-and-run’ process. These incoming EGFP-Rab7a vesicles seem to carry the signal for the maturing endosome to proceed from the initial phase to the convergence part of maturation because, in Rab7aT22N-transfected cells, endosomes never reached the convergence phase.

### Rab5-positive converged domains consist of the membrane-bound immobile fraction

We have shown that Rab5 detachment occurs in two phases, an initial rapid and a slower convergence phase after Rab7a is recruited to the endosome. Rab GTPases alternate between a membrane-bound and a cytosolic state, and the immobile and mobile fractions present on the endosomes can be measured (Sprague and McNally, 2005). We have previously described that endosome-associated proteins can specifically alter the ratio between the mobile and the long-lived immobile fraction, both as a function of signaling and between interphase and mitosis (Bergeland et al., 2008; Haugen et al., 2017). As this is believed to reflect a functional state, we wanted to investigate whether these two fractions of Rab5 on the endosomal membrane vary during the two detachment phases of Rab5.

**Fig. 5.**
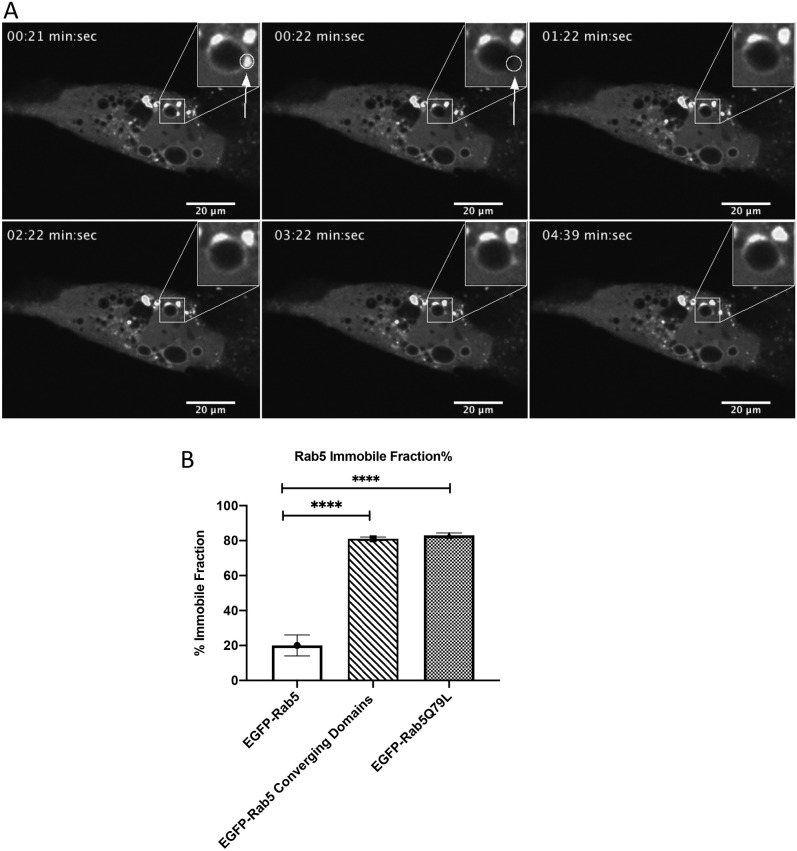
**Rab5 converged domains consist of the immobile fraction.** (A) MDCK-Ii cells transfected with EGFP-Rab5 showing enlarged endosomes positive for EGFP-Rab5 domains. One of the EGFP-Rab5-positive domains was bleached and the immobile fraction was calculated. Boxed areas are shown magnified at top right. (B) Graph showing the immobile fraction of EGFP-Rab5 (control; 20±6%, *n*=14), the immobile fraction of EGFP-Rab5 converging domain (81±1%, *n*=16) and the immobile fraction of EGFP-Rab5Q79L (83±1.4%, *n*=12). Error bars indicate ±s.d.

To analyze the binding kinetics of Rab5 before and during conversion, we performed single-endosome FRAP experiments on endosomes prior to transition as well as on converged domains. In these experiments, we used MDCK-Ii cells that expressed enlarged endosomes and had been co-transfected with EGFP-Rab5. Analysis of bleaching experiments on single endosomes positive for EGFP-Rab5 showed that EGFP-Rab5 had an immobile fraction of 22% and a mobile fraction of 78% (Fig. 5), similar to previously observed values (Haugen et al., 2017). To compare the initial Rab5 detachment with the slower convergence maturation, we had to specifically bleach the converging domains before the transfer to the newly formed Rab5-positive endosome. Enlarged endosomes together with a fast bleaching module (Andor Dragonfly mosaic) made this technically possible (Fig. 5A). Bleaching experiments and analysis of the converging domains revealed an altered binding dynamic of EGFP-Rab5 in the converged domains compared to the binding dynamics prior to domain formation (Fig. 5A). We could measure a significant change in the immobile fraction of EGFP-Rab5 in the converging domains, which increased by a factor of 4 (Fig. 5B). This result shows a gradual increase of the immobile fraction during the convergence phase prior to a complete detachment. We further showed that this immobile fraction consisted of the GTP-bound form of Rab5, by transfecting the cells with GFP-Rab5Q79L and performing the same FRAP experiment (Fig. 5B). Comparing the membrane fractions of the converging domain and GFP-Rab5Q79L we showed that, at 80% (Fig. 5B), they are the same – strongly indicating that the converging domains and Rab5 leaving the endosome to form new endosomes comprises the immobile fraction. These experiments proved that the immobile fraction is 80% in the converging domains and that this is the GTP-bound form of Rab5 (Fig. 5).

Our results show that Rab5 detached in two phases; an initial phase that may be regulated by the GDP/GTP cycle and is pertained to the mobile fraction of Rab5, and a further, slower phase that involves an increase of the Rab5-immobile fraction to converge into domains, prior to a transfer from the maturing endosome, to prime the formation of new early endosomes.

## DISCUSSION

Endosomal trafficking is carefully regulated through organelle-specific Rab GTPases (Bhuin and Roy, 2014; Zerial and McBride, 2001). These GTPases identify and regulate the progression of endocytosed macromolecules and plasma membrane receptors (Langemeyer et al., 2018). Progression and direction from the early to late endosome have been shown to be regulated by a Rab5-to-Rab7a switch (Cabrera et al., 2014; Huotari and Helenius, 2011; Nordmann et al., 2010; Poteryaev et al., 2010; Rink et al., 2005). This switch/conversion has previously been described entirely as the exchange between the cytosolic pool and the membrane-bound fraction of Rab5 and Rab7a, specifically regulated through the Rab GTP/GDP cycle (Pfeffer, 2001, 2017; Stenmark and Olkkonen, 2001).

In this article, we have shown that enlarged early endosomes in MDCK-Ii cells (Landsverk et al., 2011; Margiotta et al., 2020; Skjeldal et al., 2012; Stang and Bakke, 1997) and early endosomes in HeLa cells express transfected fluorescently tagged membrane-associated proteins, and mature into late endosomes by a stepwise Rab5 detachment that is controlled but not initiated by Rab7a. We have also shown that detachment of Rab5 follows a two-step mechanism; i.e. a fast diffusion-like initial detachment – as expected for the GTP/GDP cycle – followed by a slower process that is regulated by converging Rab5 domains (Fig. 1). When Rab5 was co-transfected with Rab7a and either wt or Q67L mutant we measured decreased Rab5 D_1/2_, indicating a regulatory role of Rab7a regarding the detachment of Rab5, specifically in the converging phase of Rab5 detachment. Furthermore, Rab7a was not recruited to the maturing endosome before the initial phase was completed, as shown in the Rab7a lag phase (Fig. 1D, blue box). This indicates that the Rab5 detachment has a Rab7a-independent and a Rab7a-dependent phase, which we confirmed after co-transfection with Rab7aT22N (dominant-negative mutant) or knocking down Rab7a by using RNAi. Under these conditions the full detachment of Rab5 did not occur and the fluorescent endosomes initiated association-/disassociation-like characteristics of Rab5 coat detachment (Fig. 2). It seemed that Rab5 detachment could not be completed because Rab7a-positive vesicles were not recruited to the endosome to initiate the converging phase of Rab5 detachment. When we expressed constitutively active Rab5 together with the dominant-negative Rab7a mutant, the specific on/off ‘blinking’ characteristics did not occur, indicating that the initial detachment of Rab5 is regulated by the activity of its GAP, i.e. independently of any possible feedback-loops later established by Rab7a.

Our experiments show that the feedback loop from the endosomal system consists of Rab7a-positive endosomes that are recruited to the Rab5-positive maturing endosome after the initial detachment of Rab5. In experiments with the dominant-negative Rab7a mutant or those of Rab7a knockdown, Rab5-positive endosomes were unable to proceed from the GTP/GDP-regulated phase into the converging phase and, consequently, the Rab5 association/disassociation pattern was observed. In between the mCh-Rab5 maximum-intensity peaks, we observed mCh-Rab5-specific domains, whose formation was abruptly terminated upon the return of uniform mCh-Rab5 coat. It seems like rab5 positive endosomes tried to reach the converging phase but lacked the signal from interacting Rab7a-positive vesicles. The ‘interacting’ Rab7a-positive endosomes provided Rab7a for the maturing endosome and, concomitantly, transmitted the signal to enter phase 2, the converging phase.

Tracking the converging phase of Rab5 we could follow the aggregation and convergence of Rab5 domains before the detachment from the maturing endosome. These Rab5-positive domains detached from the maturing endosome and give rise to newly formed Rab5-positive endosomes. Recycling of Rab5 through transfer and formation of new Rab5-positive endosomes has previously been suggested to be a possible mechanism, in addition to the Rab-GDI-mediated Rab5-to-Rab7a exchange (Rink et al., 2005). Here, we provide evidence for a direct Rab5 transfer from the maturing endosome in order to form Rab5-positive endosomes.

Furthermore, we showed that the Rab5 effector protein EEA1 follows Rab5 during convergence and transfer onto newly formed endosomes. The released endosome engaged in early endosomal fusion immediately after its formation and was able to recruit endocytosed EGF.

The membrane-bound endosomal recycling mechanism was further observed for other early endosomal proteins, such as Rab22, Rab4, Hrs and Eps15, showing that this is a general mechanism of maturation, leading to the formation of new early endosomes. Conservation of membrane-associated proteins through transfer from a maturing to a new endosome ensures a fully functional early endosome that is primed for another round of endosomal sorting and maintenance of the endosomal homeostasis.

To better understand the binding dynamics of Rab5 during the convergence phase, we performed FRAP experiments on Rab5-positive endosomes before convergence, by correspondingly bleaching the converging domains. As previously published by Sann et al. (2012), we showed that, before conversion, a Rab5-positive endosome comprised 20% immobile and 80% mobile fractions. However, in converged domains, we measured a total change in fractions, as we found 80% to be immobile and 20% to be mobile. In the control experiment, where we bleached the dominant-active mutant of Rab5 (EGFP-Rab5Q79L), we found the same distribution of immobile and mobile fractions as in converged Rab5-positive domains, which strongly suggests that the Rab5 fraction that detaches from the maturing endosome and gives rise to new endosomes is transferred as a membrane-bound GTP form.

Both EEA1 and Rab5 are tethering proteins that seem to go into a quiescent state during transition from early to late endosome, as no fusion during the Rab5-to-Rab7a exchange was observed. This may be explained by our FRAP experiment, where we measured a redistribution of Rab5-immobile and -mobile fractions. The first initial detachment proved to be the Rab5-mobile fraction that detaches during the regular GDI-mediated GTP/GDP cycle. Inactivation of the Rab5 tethering properties by redistributing the mobile to an immobile fraction may also change the EEA1 activity to inhibit the entropic collapse necessary for tethering prior to fusion (Murray et al., 2016). We hypothesize that the Rab5 fractions on the newly formed endosome are redistributed back to an 80% mobile 20% immobile fraction, in order to reactivate the fusion machinery. Redistribution of immobile and mobile fractions of Hrs and Eps15 has previously been found to be important for EGFR degradation (Haugen et al., 2017). A similar redistribution of Rab5 was shown in our current study, indicating that a localized redistribution of mobile and immobile fractions on endosomes is a general mechanism to regulate endosomal maturation and progression.

In this article we describe a new mechanism regarding the dynamics of Rab5 and Rab7a during endosomal maturation. The Rab5 to Rab7a switch is not directly an exchange in which Rab5 is replaced by Rab7a. Following the first diffusive phase, the endosomes become Rab5 and Rab7 positive (a hybrid organelle), and as the endosomal maturation progresses, we observe the formation of both Rab5 and Rab7a positive domains. Our experiments showed that the Rab5 detachment can be divided into two phases, which is similar to Rab7a recruitment. Here, we could show that the first recruitment of Rab7a is diffusive and from cytosol, where the later recruitment seems to be through fusion and ‘kiss and run'. The initial diffusive phase of Rab5 detachment may act as a switch in endosomal transition by activating the cascade of MON1A and HOPS (officially known as TMUB1) (Poteryaev et al., 2010). This is to actively recruit Rab7a to the maturing endosome, which is necessary for the convergence phase of Rab5 maturation. Formation of the initial Rab5 domains in the convergence phase might be spontaneous and GTP dependent, as found in cell-free studies (Cezanne et al., 2020). Our FRAP experiments showed a gradual increase of the relative Rab5-immobile fraction, also indicating a GTP-dependent organization of Rab5-positive domains during convergence. However, in our live cell studies, we found that formation of Rab5 domains at the later stages is Rab7a dependent. The transferred Rab5 domain is likely to be in the Rab5-GTP bound form that is able to recruit the Mon1–Ccz1 complex, a Rab7 GEF that facilitates recruitment of Rab7a (Langemeyer et al., 2020). Hence, the gradual convergence and increase of Rab5-GTP in the endosomal membrane may function as an activator of Mon1–Ccz1, in order to directly recruit Rab7a from the cytosol or to recruit Rab7a-positive vesicles to the maturing endosomes. As the Mon1–Ccz1 complex has a dual membrane targeting mechanism that binds to both Rab5 and PI-3-P, it might be the GEF/effector coupling that regulates Rab5 convergence and Rab7a recruitment during maturation (Cezanne et al., 2020; Langemeyer et al., 2020). We found that Rab7a recruitment to the endosome in transition is most likely to occur through a combination of vesicular interactions and direct recruitment from the cytosol providing Rab7a to the maturing endosome. We were unable to observe whether incoming Rab7a-positive endosomes fused with the endosome in transition or transferred from one vesicle to the other in a ‘kiss-and-run’ mechanism (Duclos et al., 2003).

The shuttling model and the maturation model both have contradictory characteristics, especially when regarding endosomal homeostasis (Helenius et al., 1983). The shuttling model predicts a set of pre-existing endosomal compartments that ferry carrier vesicles between the early and late compartments (Griffiths and Gruenberg, 1991), indicating that all early endosomes in the daughter cells pre-exist (Kamentseva et al., 2020).

Here, we have shown that early endosomes through switch from Rab5 to Rab7a, continuously giving rise to new functional endosomes – a crucial step to maintain the homeostasis of early endosomes. This maturation is regulated in a two-phased transition and provides the pool of early endosomes that exert the role as sorting organelles. Furthermore, maturation through convergence provides a direction for endosomal progression. With these novel findings, we present a crucial step in the understanding of how endosomal trafficking is regulated to maintain endosomal homeostasis and how membrane-associated proteins are recycled during endosomal maturation (see Fig. 6).

**Fig.**
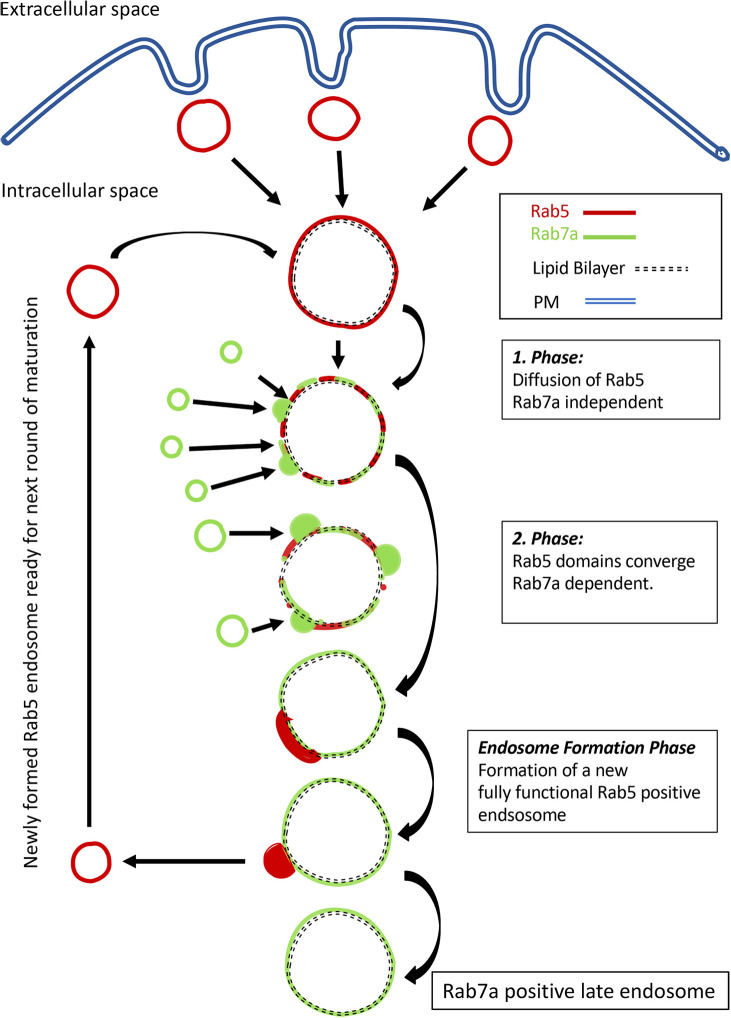
**6. Endosome maturation to endosome formation model.** The model describes the stepwise maturation pattern that gives rise to new Rab5-positive vesicles. A newly internalized Rab5-positive endosome can fuse with itself and pre-existing Rab5-positive endosomes. Maturation from Rab5- to Rab7a-positive endosomes takes place in two phases. (1) Rab7a-independent initial diffusion-like Rab5 detachment. (2) Convergence phase, during which Rab5-positive domains are formed and converge towards one main domain. This phase is dependent on Rab7a-positive endosome interaction to provide and/or supply Rab7a to the maturing endosome. Finally, the converged domains leave the matured endosome to generate new and fully functional Rab5-positive endosomes that will be primed for the next round of endosomal sorting through maturation (see main article for detailed description).

## MATERIALS AND METHODS

### Cell culture

Madine–Darby canine kidney strain II (MDCK) and HeLa cells were grown in an incubator at 37°C and 5% CO_2_ in complete Dulbecco's modified Eagle's medium (DMEM; Bio Whittaker), supplemented with 10% fetal calf serum (FCS; Integro), 2 mM L-glutamine and 25 U/ml penicillin (all from Bio Whittaker). MDCK cells, stably transfected with invariant chain (Ii) in the pMep4 plasmid, were grown under the same conditions, additionally supplemented with 25 µg/ml hygromycin (Bio Whittaker).

### DNA constructs

The cDNA encoding Ii (CD74) has been previously described (Bakke and Dobberstein, 1990). Subcloning and use of the Ii fragment into the pMep4 plasmid has been previously described (Gregers et al., 2003; Skjeldal et al., 2012). The plasmid encoding mCherry-Rab5 (mCh-Rab5) has been previously described (Haugen et al., 2017). EGFP-Rab5Q79L was generously provided by Mitsonuri Fukuda (Tohoku University, Miyagi, Japan). pEGFP-Rab7a, pEGFP-Rab7aQ69L and pEGFP-Rab7aT22N were acquired from Cecilia Bucci's laboratory (University of Salento, Lecce, Italy) and have been described previously (Bucci et al., 2000). The plasmid encoding dsRed-Rab7aT22N was a gift from Richard Pagano (Mayo Clinic and Foundation, Rochester, USA), through Addgene (plasmid #12662). The plasmid encoding mApple-Rab7a (Addgene plasmid #54945) was a gift from Michael Davidson (Florida State University). EGFP-Rab22 was purchased from Addgene (plasmid #49600). The plasmids encoding mRFP-Hrs and EGFP-Eps have been previously described by Haugen et al. (2017). EGFP-Rab4A (plasmid #49434) was purchased from Addgene.

### Cell transfection

MDCK or HeLa cells were seeded in 6-well plates or 35 mm glass-bottom Petri dishes (MatTek Corporation). Transfections were performed overnight using Lipofectamine^®^ 2000 (Invitrogen), according to the manufacturer's instructions. For each transfection, ≤1 µg DNA was used. Cells were imaged 1 day after transfection.

### Expression of Ii (MDCK) and uptake of EGF (HeLa)

MDCK cells stably transfected with Ii-pMep4 were grown in 35 mm glass-bottom Petri dishes (MatTek), in the presence of complete DMEM. To induce the expression of Ii, the cells were incubated with 25 µM CdCl_2_ overnight. Before imaging, the cells were washed with PBS. EGF-Alexa-Fluor-647 (Molecular Probes) was added to the HeLa cells, on the microscope, at a final concentration of 40 ng/ml.

### RNA interference

RNA interference (RNAi) was used to knock down Rab7a in HeLa cells. Cells were plated 1 day before transfection in complete DMEM. Transfection was done with Oligofectamine™ (Invitrogen) and by using 90 nM small interfering RNA (siRNA) targeting Rab7a or a non-targeting sequence for negative control. The following oligonucleotide sequences were used: Rab7a siRNA sense sequence 5′-GGAUGACCUCUAGGUCAUCC-3′, antisense sequence 5′-UUCUUCCUAGAGGUCAUCC-3′ (Spinosa et al., 2008).

### Western blotting and antibodies

Western blotting was used to determine the efficiency of the Rab7a knockdown. The silenced and control cells were lysed in buffer containing 25 mmol HEPES, 125 mmol C_2_H_3_KO_2_, 2.5 mmol Mg(C_2_H_3_O_2_)_2_, and 5 mmol EGTA. Immediately before lysis, the buffer was complemented with 1 mM DTT, 0.5% NP-40 and a protease inhibitor cocktail (Roche). 20 µg of protein were loaded on pre-cast Tris-HEPES gels (NuSep 12%) and transferred to Immobilon-P PVDF membranes (Millipore). The PVDF membranes were blotted with specific antibodies dissolved in 2% milk solution overnight at 4°C. Bands were visualized after incubation (ECL chemiluminescence kit; Amersham, GE Healthcare). Anti-tubulin antibody was used as a loading control (monoclonal mouse anti-α-tubulin, Zymed, Thermo Fisher Scientific; dilution (1:10000). Primary Rab7a antibody was purchased from Cell Signaling Technology (#2094S), and used at a dilution of 1:500. HRP-linked donkey anti-rabbit antibody (NA934 GE Healthcare LifeSciences) was used as secondary antibody. Relative protein levels were measured using ImageJ.

### Live cell imaging and FRAP experiments

Live cell experiments were performed on four different microscopes; 1: Olympus iX81 FluoView 1000 inverted confocal microscope (Olympus, Hamburg, DE), equipped with a PlanApo 60×1.30 oil objective. 2: Olympus SpinSR10 spinning disk confocal super resolution microscope equipped with a Yokogawa CSU-W1 SoRa. Images are acquired with a 60× Plan Apo 1.42 NA objective. 3: Zeiss LSM 880 equipped with and Airyscan detector and image acquisition with a 63×1.40NA oil immersion objective. 4: The FRAP experiments were executed on an Andor Dragonfly equipped with a Mosaic FRAP module. The Andor Dragonfly is built on a Nikon TiE inverted microscope equipped with a 60×1.40 NA oil immersion objective. Bleaching of the EGFP-Rab5 was done with 405 nm laser. Bleaching experiments were done by using 100% laser power, 100 ms bleaching time for the full-size endosome and 20 ms for converged Rab5 domains.

Data obtained from FRAP were normalized and corrected for bleaching (Pelkmans et al., 2001) and fitted by nonlinear regression to a function that assumes a single diffusion coefficient (Yguerabide et al., 1982);(1)
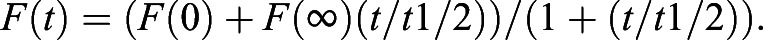
The values for *F*(0), *F*(∞) and *t*_1/2_ were calculated using GraphPad Prism 8, and immobile fractions (IF) were calculated as described by Lippincott-Schwartz et al. (2001).

### Single-endosome analysis and quantification of D_1/2_

Single Rab5- and Rab7a-positive endosomes in MDCK-Ii and HeLa cells were manually tracked using ImageJ, and mean intensity was measured over time. The results from 12–16 single endosomes per experiment were averaged over time with the variation depicted as the ±standard deviation (±s.d.) in the graphs. D_1/2_ detachment was calculated by performing a non-linear regression fit (Prism 8).

## Supplementary Material

Supplementary information

Reviewer comments
