# Supplementary material for: De novo formation of early endosomes during Rab5-to-Rab7a transition
Source: J Cell Sci. 2021 Apr 27;134(8):jcs254185. doi: 10.1242/jcs.254185 (PMC8106955; doi:10.1242/jcs.254185)
Supplement: Supplementary information [file joces-134-254185-s1.pdf]

Fig S1

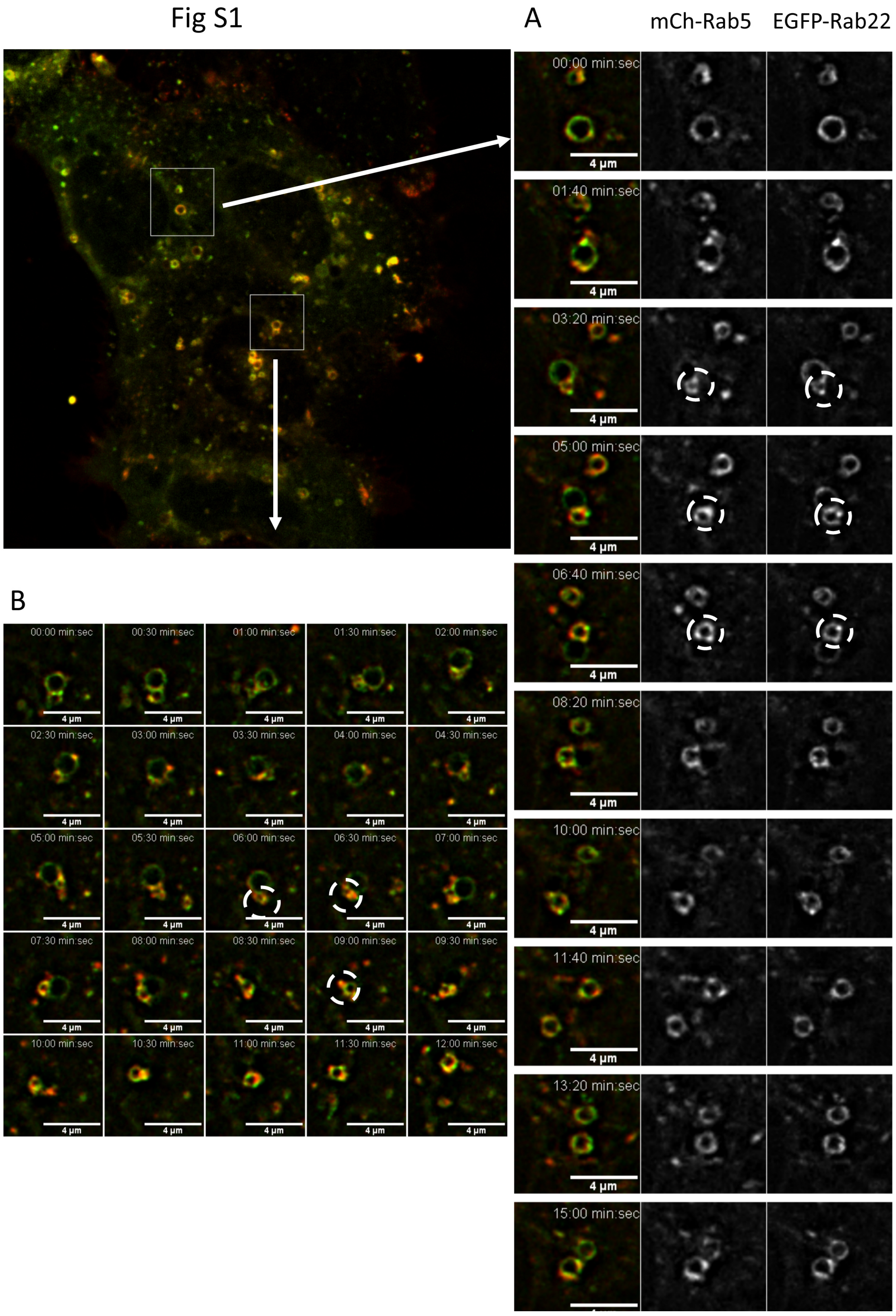

Journal of Cell Science • Supplementary information

Fig. S1 A and B:

Hela cells expressing mCh-Rab5 and EGFP-Rab22 localized to early endosomes. The image sequence shows a deconvolved maximum projection where we can follow the gradual detachment of mCh-Rab5 and EGFP-Rab22 to the formation of a new mCh-Rab5 and EGFP-Rab22 positive endosome (white rings).

Journal of Cell Science • Supplementary information

Fig S2:

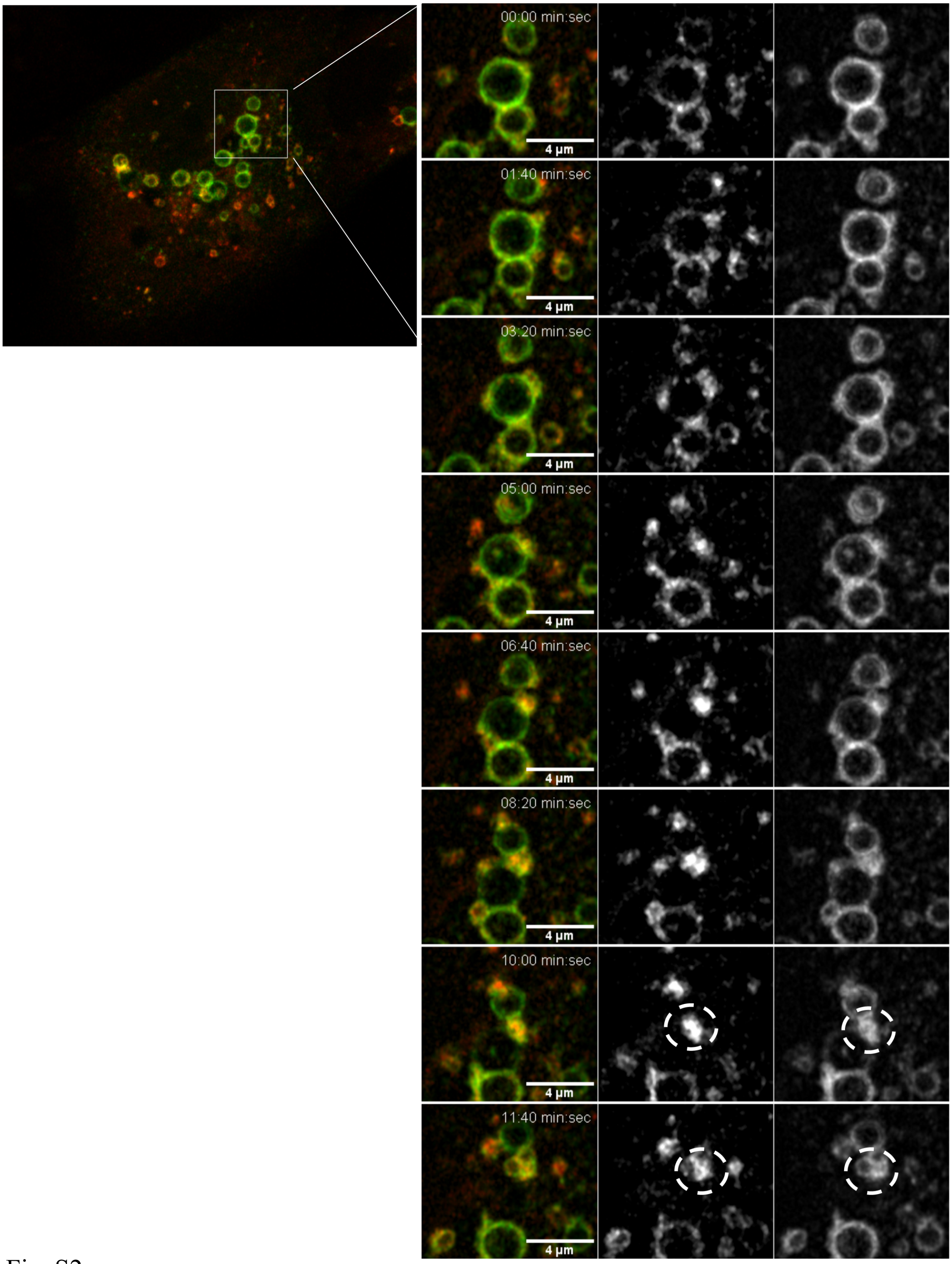

Fig. S2:

MDCK-Ii cells expressing mCh-Rab5 and EGFP-Rab22 localized to early endosomes. The image sequence shows a deconvolved maximum projection where we can follow the gradual detachment of mCh-Rab5 and EGFP-Rab22 to the formation of a new mCh-Rab5 and EGFP-Rab22 positive endosome (white rings).

Journal of Cell Science • Supplementary information

Fig S3:

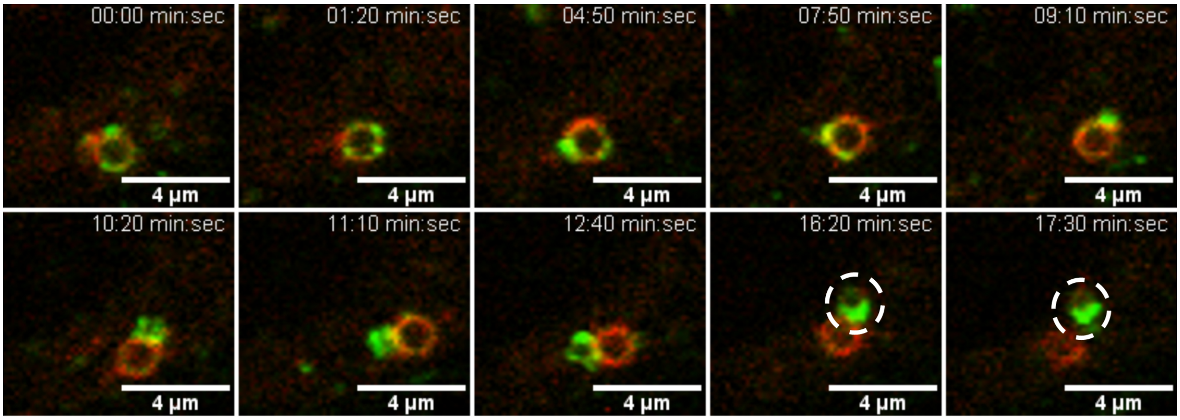

Fig. S3:

Hela cells expressing mApple-Rab7 and EGFP-Rab22 localized to early endosomes. The image sequence shows a deconvolved maximum projection where we can follow the gradual transition from a EGFP-Rab22 positive endosome to a mApple-Rab7 positive endosome. During this transition we can observe the formation of a new EGFP-Rab22 positive vesicle (white ring).

Fig S4:

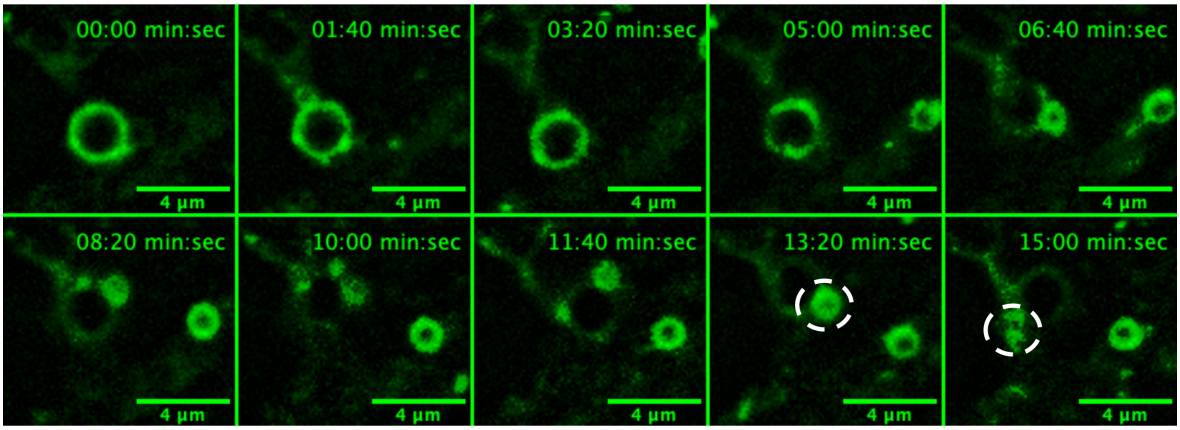

Fig. S4:

Hela cells expressing EGFP-Rab4a localized to early endosomes. The image sequence shows a deconvolved maximum projection where we can follow the gradual convergence of EGFP-Rab4a during maturation, to form a new EGFP-Rab4a positive endosome.

Journal of Cell Science • Supplementary information

Fig S5:

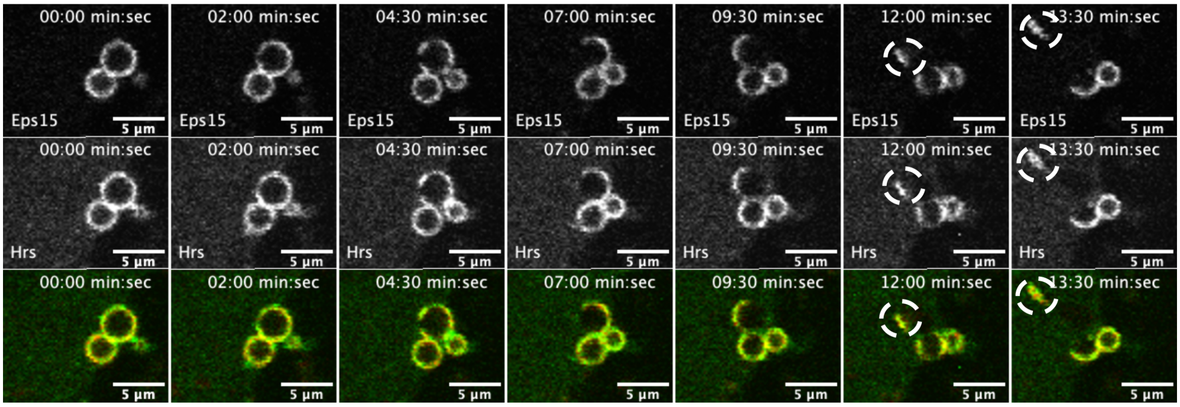

Fig. S5:

MDCK-Ii cells expressing mRFP-Hrs and EGFP-Eps15 localized to early endosomes. The image sequence shows the gradual detachment of mRFP-Hrs and EGFP-Eps15 to the formation of a new mRFP-Hrs and EGFP-Eps15 positive endosome (white rings).

Journal of Cell Science • Supplementary information

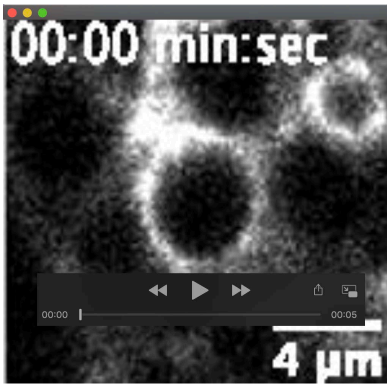

Movie 1:
MDCK-Ii cells co-transfected with mCh-Rab5. This movie demonstrates the gradual detachment of mCh-Rab5 from the endosomal membrane. Fig. 1A movie.

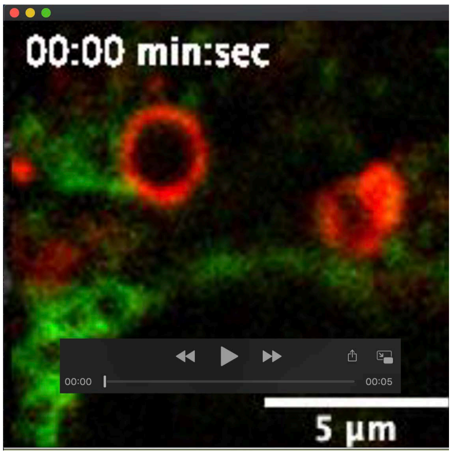

Movie 2:
MDCK-Ii cells co-transfected with mCh-Rab5 and EGFP-Rab7a. This movie demonstrates the gradual exchange from mCh-Rab5 to EGFP-Rab7a on two endosomes. Fig. 1B movie.

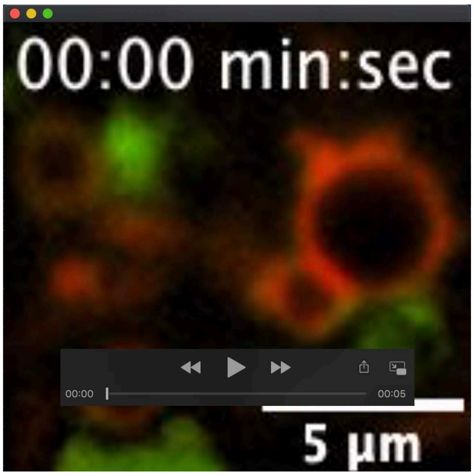

Movie 3:
MDCK-Ii cells co-transfected with mCh-Rab5 and EGFP-Rab7aQ67L. This movie demonstrates the exchange between mCh-Rab5 and mutant EGFP-Rab7aQ67L. Fig. 1C movie.

Journal of Cell Science • Supplementary information

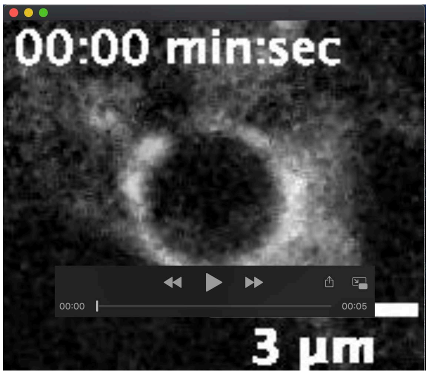

Movie 4:
HeLa cells transfected with mCh-Rab5 and Rab7T22N. This movie demonstrates the detachment and reattachment of mCh-Rab5. mCh-Rab5 positive endosomes are unable to fully detach due to the lack of Rab7 recruited to the maturing endosome. Fig.2B movie.

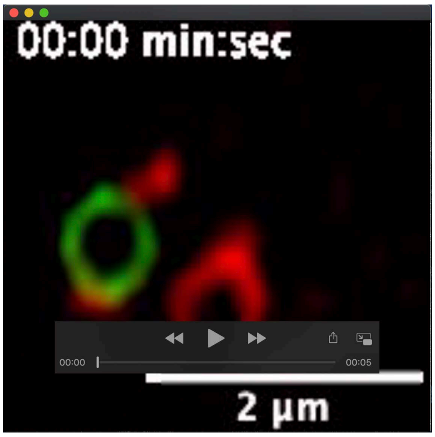

Movie 5:
HeLa cells transfected with EGFP-Rab5 and mApple-Rab7a. The movie demonstrates the gradual exchange between EGFP-Rab5 and mApple-Rab7a and shows specifically the convergence of microdomains that ends up with the formation of a novel EGFP-Rab5 positive endosome. Fig. 3B movie

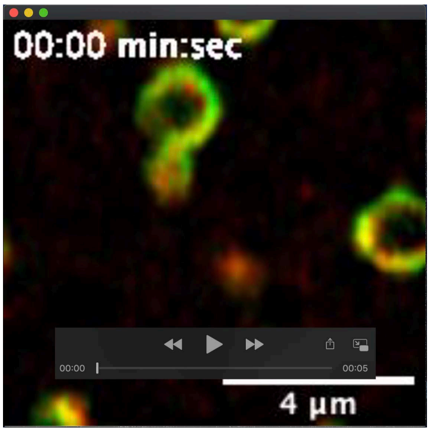

Movie 6:
HeLa cells transfected with EEA1-GFP and mCh-Rab5. This movie demonstrates a similar detachment pattern as described in 3B. However, here we can also follow EEA1-GFP in the formation of a novel early endosome. Fig. 3C movie.

Journal of Cell Science • Supplementary information

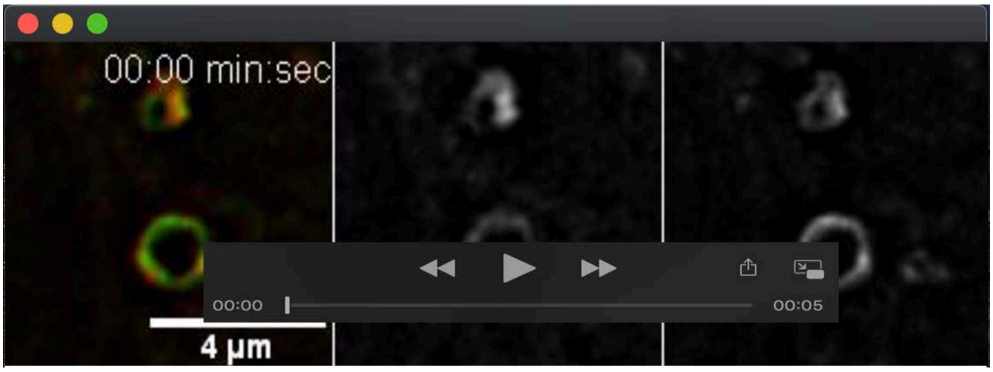

Movie 7:
HeLa cells transfected with mCh-Rab5 and EGFP-Rab22 showing endosomal convergence to formation of a new endosomes. This is a deconvolved maximum projection of 3 confocal planes per timepoint with a stepsize of 0.32 µm distance. Fig. S1A movie.

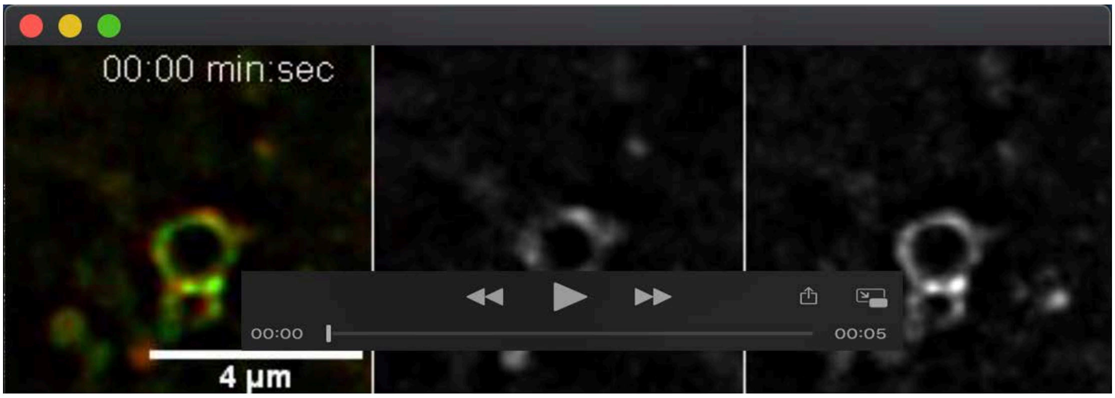

Movie 8:
HeLa cells transfected with mCh-Rab5 and EGFP-Rab22 showing endosomal convergence to formation of a new endosomes. This is a deconvolved maximum projection of 3 confocal planes per timepoint with a stepsize of 0.32 µm distance. Fig. S1B movie.

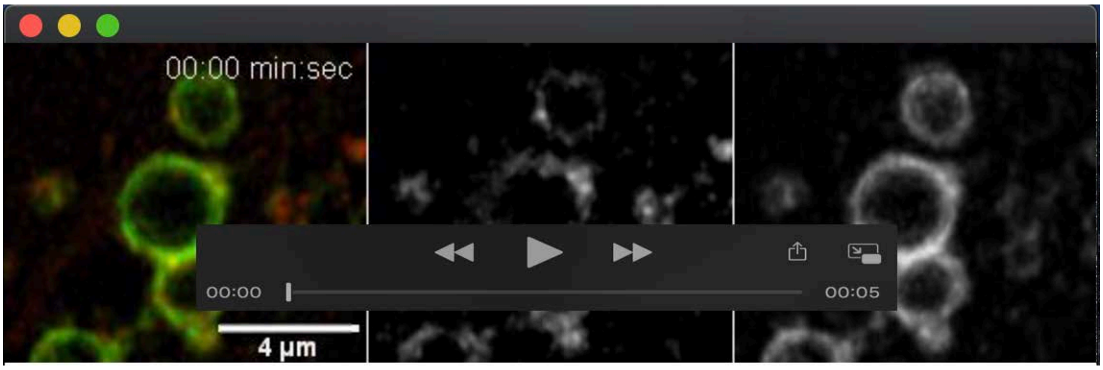

Movie 9:
MDCK-Ii cells transfected with mCh-Rab5 and EGFP-Rab22 showing the same endosomal convergence to formation of a new endosome. This is a deconvolved maximum projection of 5 confocal planes per timepoint with a stepsize of 0.32 µm distance. Fig. S2 movie.

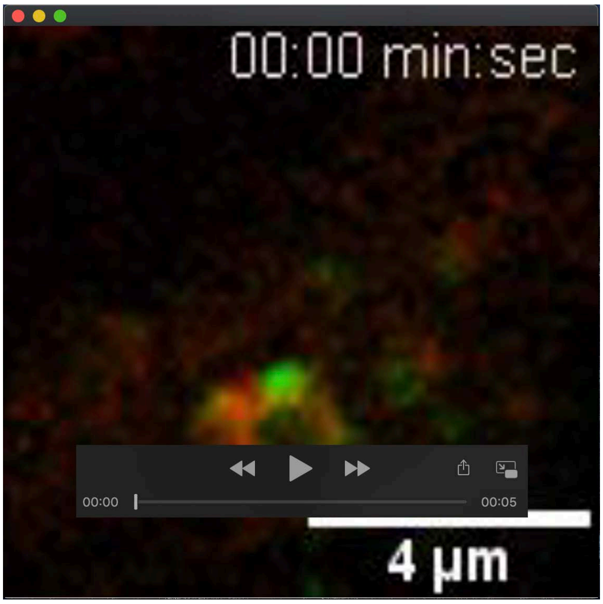

Movie 10:
HeLa cells transfected with EGFP-Rab22 and mCh-Rab7a showing the same endosomal convergence to formation of a new endosome. This is a deconvolved maximum projection of 3 confocal planes per timepoint with a stepsize of 0.32 µm distance. Fig. S3 movie.

Journal of Cell Science • Supplementary information

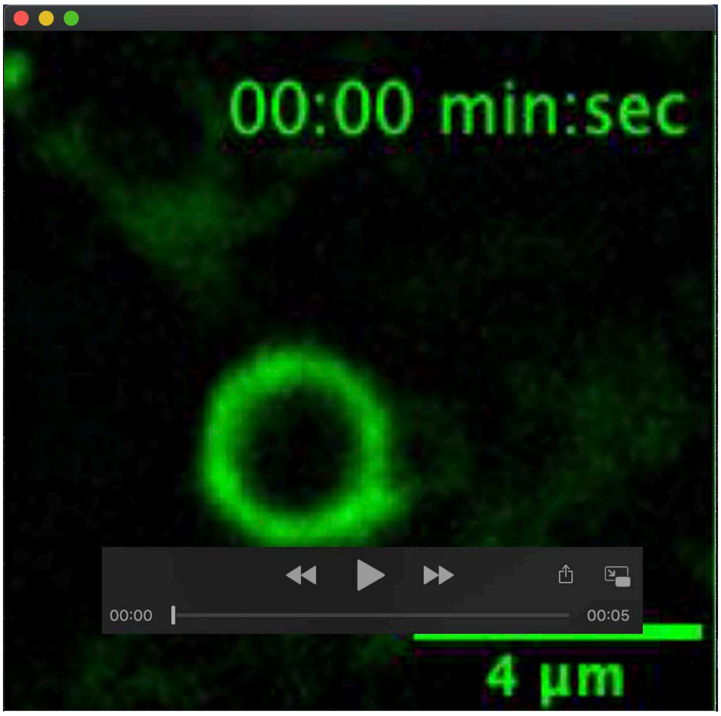

Movie 11:
HeLa cells transfected with EGFP-Rab4a showing the same endosomal convergence to formation of a new endosome. This is a deconvolved maximum projection of 3 confocal planes per timepoint with a stepsize of 0.32 µm distance. Fig. S4 movie.

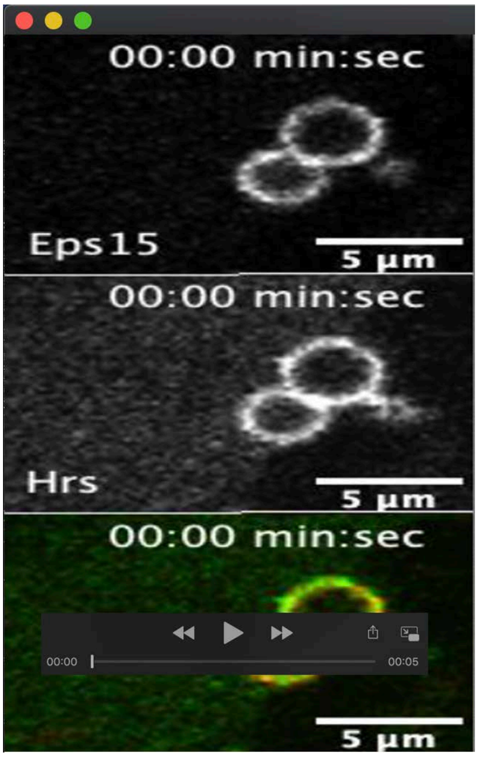

Movie 12:
MDCK-Ii cells transfected with mRFP-Hrs and EGFP-Eps15 showing the same endosomal convergence to formation of a new endosome. Fig. S5 movie.

Journal of Cell Science • Supplementary information

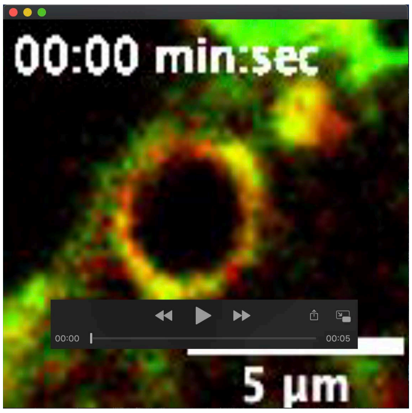

Movie 13:
MDCK-Ii cells co-transfected with mCh-Rab5 and EGFP-Rab7a. This movie demonstrates the influence of EGFP-Rab7a positive vesicle delivering the necessary EGFP-Rab7a to the maturing endosome. EGFP-Rab7a vesicular recruitment to maturing endosomes. Fig. 4A movie.

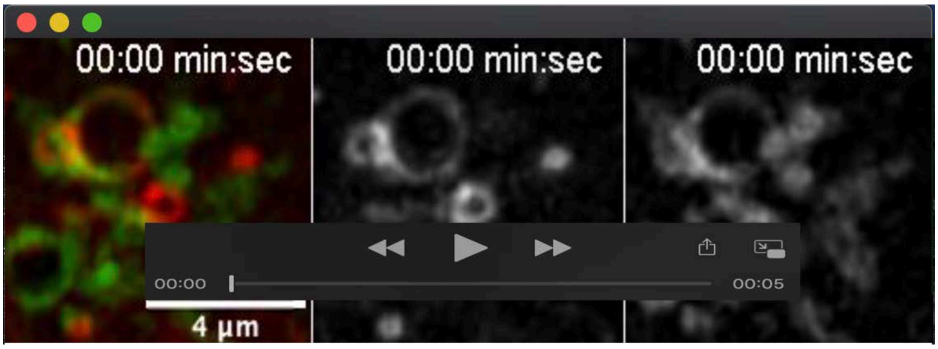

Movie 14:
HeLa cells transfected with mCh-Rab5 and EGFP-Rab7a. This is deconvolved maximum projection of 3 confocal planes per timepoint with a stepsize of 0.32 µm distance. The movie demonstrates mCh-Rab5 convergence maturation together with an EGFP-Rab7a recruitment during the Rab5-Rab7 transition. Fig. 4B movie.

Journal of Cell Science • Supplementary information

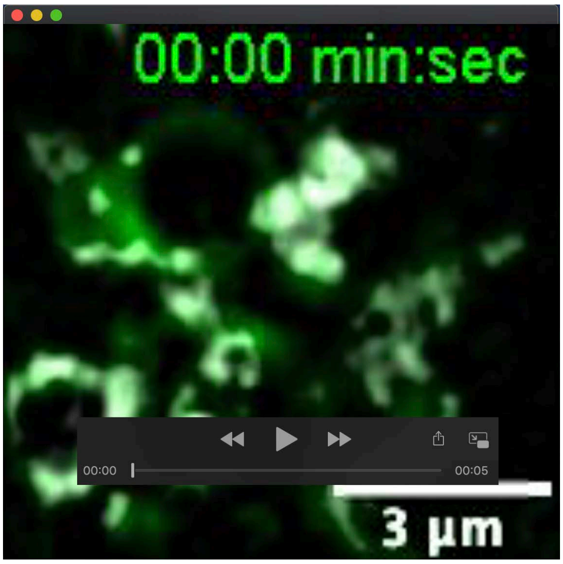

Movie 15:
This movie shows the recruitment of EGFP-Rab7a to the maturing endosome by vesicular interactions. mCh-Rab5 signal has been subtracted from EGFP-Rab7 signal to remove any overlapping signal on the hybrid endosome. By this method we can better visualize the contribution of the EGFP-Rab7a interacting vesicles (in white) compared to the original EGFP-Rab7a signal (in green). Fig. 4C movie.

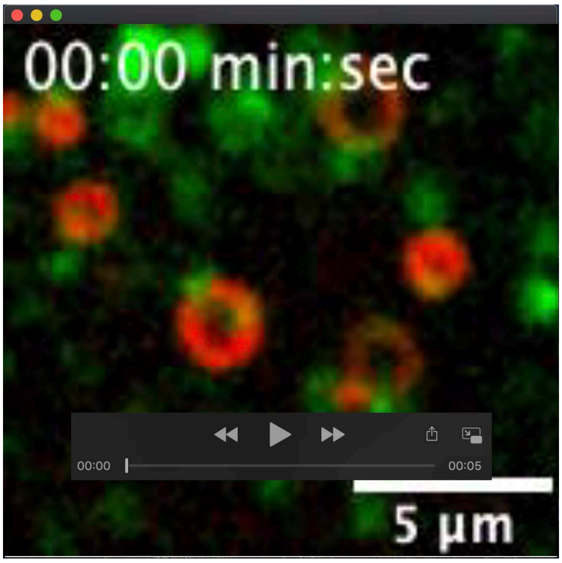

Movie 16:
MDCK-Ii cells transfected with EEA1-mRFP and EGFP-Rab7a. This movie demonstrates similar EGFP-Rab7a vesicular delivery to EEA1-mRFP positive vesicles.
Maturing endosomes recruits Rab7 positive vesicles during maturation. Fig. 4D movie.
