# Supplementary material for: De novo formation of early endosomes during Rab5-to-Rab7a transition
Source: J Cell Sci. 2021 Apr 27;134(8):jcs254185. doi: 10.1242/jcs.254185 (PMC8106955; doi:10.1242/jcs.254185)
Supplement: Reviewer comments [file joces-134-254185_review_history.pdf]

De novo formation of early endosomes during Rab5-to-Rab7a transition

Frode Miltzow Skjeldal, Linda Hofstad Haugen, Duarte Mateus, Dominik M. Frei, Anna Vik Rødseth, Xian Hu and Oddmund Bakke
DOI: 10.1242/jcs.254185

Editor: Jennifer Lippincott-Schwartz

Review timeline

Original submission:	14 September 2020
Editorial decision:	26 October 2020
First revision received:	6 January 2021
Editorial decision:	4 February 2021
Second revision received:	11 February 2021
Accepted:	18 February 2021

Original submission

<u>First decision letter</u>

MS ID#: JOCES/2020/254185

MS TITLE: De novo formation of early endosomes during Rab5 to Rab7 transition

AUTHORS: Oddmund Bakke, Frode M Skjeldal, Linda Haugen, Duarte Mateus, and Dominik Frei
ARTICLE TYPE: Research Article

We have now reached a decision on the above manuscript.

To see the reviewers' reports and a copy of this decision letter, please go to: https://submit-jcs.biologists.org and click on the 'Manuscripts with Decisions' queue in the Author Area. (Corresponding author only has access to reviews.)

As you will see, the reviewers gave favourable reports but raised some critical points that will require amendments to your manuscript. I hope that you will be able to carry these out, because I would like to be able to accept your paper.

We are aware that you may be experiencing disruption to the normal running of your lab that makes experimental revisions challenging. If it would be helpful, we encourage you to contact us to discuss your revision in greater detail. Please send us a point-by-point response indicating where you are able to address concerns raised (either experimentally or by changes to the text) and where you will not be able to do so within the normal timeframe of a revision. We will then provide further guidance. Please also note that we are happy to extend revision timeframes as necessary.

Please ensure that you clearly highlight all changes made in the revised manuscript. Please avoid using 'Tracked changes' in Word files as these are lost in PDF conversion.

I should be grateful if you would also provide a point-by-point response detailing how you have dealt with the points raised by the reviewers in the 'Response to Reviewers' box. Please attend to all of the reviewers' comments. If you do not agree with any of their criticisms or suggestions please explain clearly why this is so.

© 2021. Published by The Company of Biologists under the terms of the Creative Commons Attribution License (https://creativecommons.org/licenses/by/4.0/).

Reviewer 1

Advance summary and potential significance to field

This paper by Skjeldal and coworkers provides evidence for the segregation and removal by budding of Rab5 from early endosomes in a late step of endosomal maturation. They also propose that the arrival of Rab7 to early endosomes may be in part delivered by fusion of Rab7-positive vesicles. This contrasts with models developed by the Zerial lab and others in which arrival of Rab5 and exchange of Rab5 with Rab7 can be explained entirely through factors exchanging between the endosomal surface and cytosol. While the models proposed in the current paper are intended as a supplement to the existing model rather than a replacement, they raise a series of interesting questions likely to inspire further research in this area.
However, there are some serious technical issues in the work that need to be addressed..

Comments for the author

Major issues.

1. The distribution of Rab proteins on large spherical endosomes are examined by viewing single confocal sections through the centers of the endosomes. Thus, a large fraction of the GFP-Rab associated with the endosome is invisible. I think this is not an issue in some quantitative expts (e.g. Figure 1) where data from many endosomes is pooled. However, the core experiments of the paper, purporting to show consolidation of Rab5 into a single region, and the removal of the Rab5 region as a budding vesicle suffer from the same limitations. This is a major novel conclusion of the paper which needs additional work to be convincing. E.g., in Movie 3b, it seems difficult to rule out that a second Rab5 positive endosome moved vertically while being accidently aligned with the first endosome. It is stated that this is an invariant outcome, which if true, would argue against coincidence. Some examples are shown (e.g., Figure 3a, Figure 3b). To back such a strong statement, there needs to be a count of a reasonable number of endosomes (e.g. 20 or so) and a statement of the proportion of cases in which consolidation and removal of Rab5 happens, with the backing data ideally presented as a supplement in a format similar to Figure 3b. Ideally, the optical slicing issue should be addressed by repeated z-section, but this may not be technically possible at the time intervals required, so the limitations of the data need to be discussed.

2. Related to #1, given the uneven distribution of GFP-Rab around the margins of some endosomes, the oscillations in Rab5 levels reported in Figure 2 could possibly represent vertical movement, or even rotations of the endosomes rather than fluctuations in the levels of Rab5. Since the endosomes are large this could probably be addressed with non-confocal images taken at lower resolution to avoid optical slicing.

3. I'm skeptical of statements that Rab7-positive endosomes cluster around Rab5-positive endosomes prior to maturation, simply because of the high density of both kinds of endosomes (as seen in the movies shown). A requirement for introduction of Rab7 into EE by vesicle fusion would, of course, be of major scientific interest if true. However, showing a convincing and biologically relevant effect would require some non-trivial quantitation and statistical analysis, and also demonstration of at least transient fusion of Rab7 endosomes with the EE, which I think would go beyond the scope of the current paper. The authors should be clear on the limitations of the current data, and should also cite and discuss a new paper (Langemeyer et al (2020) Elife 9:e56090) which makes an argument that, at least in an in vitro system conversion from Rab5 to Rab7 can take place entirely through the actions of cytosolic factors and factors associated with the EE membrane.

4. The kinetic analyses in the paper follow visible (GFP-tagged) Rab5 and Rab7. This is difficult to avoid but the authors need to make clear to the readers that they are not following the invisible pool of untagged endogenous Rab5/Rab7, and that there could be kinetic or other differences. This is particularly important with respect to the interesting observation made in the paper that Rab5 levels drop by about 50% prior to the initial arrival of tagged Rab7.

© 2021. Published by The Company of Biologists under the terms of the Creative Commons Attribution License (https://creativecommons.org/licenses/by/4.0/).

5. The significance of the removal of the final pool of Rab5 in a vesicle depends in part on whether this vesicle is important to seed Rab5 into new early endosomes. Given the positive feedback loop that maintains Rab5 on the membrane, this is plausible. However, they need to acknowledge and discuss previously published papers (e.g., Cezanne (2020) Elife 9:354434) which propose other mechanisms, e.g. ubiquitin binding, which have been proposed for recruitment of the initial Rab5.

Minor:

1) In the discussion, there is a paragraph regarding properties of endosomes during mitosis. The relevance of this needs to be clarified. Is endosomal formation/maturation frozen or otherwise modified during mitosis?

2) The statement in the discussion that "Both EEA1 and Rab5 are fusogenic membrane associated proteins . . ." needs modification. Both play a role in regulating membrane fusion, but, unlike SNARES and the spike proteins of enveloped viruses, are not capable of driving fusion themselves.

Reviewer 2

Advance summary and potential significance to field

The manuscript of Skjeldal et al. follow in their study the behaviour of Rab5 and Rab7 during endosomal maturation. The authors use transfection with fluorophore-labeled Rab5 and Rab7 Rabs either without mutations, or with mutations favouring the GTP or GDP/empty conformation of the Rab and trace their localization relative to each other over time. As they use cells expressing the invariant chain, these cells have enlarged endosomes, facilitating their analysis of relative localization. The authors then find evidence that Rab5 detaches in domains from these enlarged endosomes before Rab7 is found on endosomes, and Rab7 thus does not seem to be required for this process. It thus seems that Rab5 positive endosomes emerge during this transition in a budding process. They also find evidence for a step-wise transition of this process.

Comments for the author

The authors rely in their study primarily on GFP or RFP-tagged Rab5 and Rab7, which are apparently overexpressed in cells. Their fluorescence microscopy looks well done and controlled and indeed shows a striking transition from endosome-associated Rab5 to Rab5 domains and Rab5-positive and Rab7 negative vesicles. This apparent vesicular exchange is quite different from the model, in which Rab7 recruitment and Rab5 release are coupled (Rink et al., 2005, Poterayev et al, 2010). My problem with the study is rather that I am not sure what I really learn from this. In all cells, the wild-type protein is still present. If the authors really want to make a point in the vesicle exchange of Rab5 and Rab7, then they need to interfere with the endogenous protein by interfering with the Rab7 GEF or the Rab5 GAP, or by inactivating the endogenous Rab5 and expressing an siRNA-resistant Rab5 instead. Of course, tracing Rab5 and Rab7 has a value of its own as the extra copies of each protein may reflect the movement of endosomal membranes, yet the authors do not trace membrane per se in their assays, but the fluorophore-labelled structures and apparently do strong overexpression. At this point, this is an interesting observation, where I miss a lot of controls. I will list some of my concerns that may help them to frame the study better.

1. The study starts out with a misunderstanding of the Rab7 GEF, which is a bit unfortunate. Several studies showed that the Mon1-Ccz1 complex is the Rab7 GEF in yeast, Drosophila, plants and humans, a crystal structure is available of Mon1-Ccz1 and Rab7, and a recent study showed clearly that Rab5 is required for Rab7 activation (Langemeyer et al., 2020). Also Nordmann et al., 2010 showed already that Vps39 has no GEF activity. This needs to be corrected in their introduction and discussion part.
2. The authors describe a two-phase release process (Figure 1C). They should repeat the experiment in cells, where they silenced the Mon1-Ccz1 GEF complex and follow Rab7 in parallel. I expect that the Rab7 is then primarily in the cytosol, which would be a good control for their assays.

© 2021. Published by The Company of Biologists under the terms of the Creative Commons Attribution License (https://creativecommons.org/licenses/by/4.0/).

3. Along this line, the authors mention that Rab7 is not required for Rab5 release. However, they still have the wild-type protein in the background, and just following the overexpressed Rab7 does not really make this point.
4. Figure 1 – graphs in A-D. The authors label only the Y-axis with the mCherry Rab5, but also trace Rab7 (GFP-tagged), and this has to be added as an information.
5. Figure 3D is missing, and I can thus comment only on the text. In fact, I am not sure what to learn from this. Does this mean that EGFR is recycled once taken up into the cell? I always thought that EGFR moves directly to the MVB and the lysosome?
6. In Figure 3C and F, the authors trace EEA1. As EEA1 binds Rab5-GTP, this is one of the few cases, where an apparent cargo and thus membrane is followed. Based on this, the Rab5-positive structures contain active Rab5, indicating indeed that Rab5 is not inactivated when Rab7 arrives. Previous work indicates that Rab5 and Rab7 bind also retromer. It would be of interest if the authors repeat their analysis when they inactivate retromer as the separation of endosomal domains likely requires this complex.
7. I find it important that the authors trace the identity of their membranes with additional markers beyond Rab5 and Rab7 such as the FYVE domain to get additional information on its identity.

First revision

Author response to reviewers' comments

Response to reviewers:
Thanks to the reviewers for the constructive criticism on our work submitted to JCS. We found the constructive criticism very stimulating and helpful for improving the quality of our current work. The feedback from the reviewers additionally opened up some new ideas for our future work to understand the molecular mechanism of the convergence maturation.
We hereby submit a new modified version to meet the evaluation from the reviewers. A general concern from the reviewers addressed the number of experiments showing the same type of maturation pattern. We have now added two more figures (Figure 3F and G) and five supplementary figures. All of these new figures come with a supplementary movie emphasizing the main findings of the paper. The additional figures and movies are all maximum projections of 4D movies to meet the technical request from the reviewers concerning "hidden" information above or below the confocal plane.
We have furthermore rewritten parts of the text and discussed the main findings in the paper with regards to the suggested newly published papers in eLife, as suggested by the reviewers. This input enabled us to add important information to the discussion.
We would like that the reviewers notice the convergence of Rab5 domains in all the figures, and domains of the 4 additional membrane associated proteins presents in this paper. We have hundreds of similar movies, also in other cell lines, but of course we have to show only a few in a paper.

Response to reviewer 1:
Reviewer 1 Advance Summary and Potential Significance to Field:
This paper by Skjeldal and coworkers provides evidence for the segregation and removal by budding of Rab5 from early endosomes in a late step of endosomal maturation. They also propose that the arrival of Rab7 to early endosomes may be in part delivered by fusion of Rab7-positive vesicles. This contrasts with models developed by the Zerial lab and others in which arrival of Rab5 and exchange of Rab5 with Rab7 can be explained entirely through factors exchanging between the endosomal surface and cytosol. While the models proposed in the current paper are intended as a supplement to the existing model rather than a replacement, they raise a series of interesting questions likely to inspire further research in this area. However, there are some serious technical issues in the work that need to be addressed.

Reviewer 1 Comments for the Author:
Major issues.

© 2021. Published by The Company of Biologists under the terms of the Creative Commons Attribution License (https://creativecommons.org/licenses/by/4.0/).

1. The distribution of Rab proteins on large spherical endosomes are examined by viewing single confocal sections through the centers of the endosomes. Thus, a large fraction of the GFP-Rab associated with the endosome is invisible. I think this is not an issue in some quantitative expts (e.g. Figure 1) where data from many endosomes is pooled. However, the core experiments of the paper, purporting to show consolidation of Rab5 into a single region, and the removal of the Rab5 region as a budding vesicle suffer from the same limitations. This is a major novel conclusion of the paper which needs additional work to be convincing. E.g., in Movie 3b, it seems difficult to rule out that a second Rab5 positive endosome moved vertically while being accidently aligned with the first endosome.

The point being made by the reviewer here is very important to address and actually concerns most imaging studies today. In an ideal imaging experiment, we would like acquire every timeframe in 3D and with as many timeframes as possible. However, due to bleaching and phototoxicity this is very challenging to achieve with today's technology. For this revised version we have performed experiments with 3-8 z-sections to achieve a thicker section and you will find in the new figures as maximum projections to include more info into the micrographs. These new experiments with thicker illuminated sections provide the same results and supports the conclusions already made. All the supplementary figures are maximum projections to improve the data as requested by the reviewer's comments.

"it seems difficult to rule out that a second Rab5 positive endosome moved vertically while being accidently aligned with the first endosome".
It is stated that this is an invariant outcome, which if true, would argue against coincidence. Some examples are shown (e.g., Figure 3a, Figure 3b). To back such a strong statement, there needs to be a count of a reasonable number of endosomes (e.g. 20 or so) and a statement of the proportion of cases in which consolidation and removal of Rab5 happens, with the backing data ideally presented as a supplement in a format similar to Figure 3b. Ideally, the optical slicing issue should be addressed by repeated z-section, but this may not be technically possible at the time intervals required, so the limitations of the data need to be discussed.

To rule out that this is an accidental event we have observed hundreds of endosomes and all show the same convergence to a new endosome formation. From the analysis performed in Fig. 1 we find more than 90% of the endosomes measured matures through convergence. This we have stated in the new version. To support our findings, we have added 5 supplementary figures and videos that show the exact same conversion and transfer and we also use other membrane associated proteins. The new supplementary figures, show that Rab22, Rab4, Hrs and Eps15 follow to the nascent endosome like Rab5, first convergence and then transfer.
To conclude, this convergence maturation and the transfer of early endosomal associated protein to induce the formation of an early endosome seems to be a general mechanism to transfer the necessary membrane associated proteins to fully functional early endosomes.

2. Related to #1, given the uneven distribution of GFP-Rab around the margins of some endosomes, the oscillations in Rab5 levels reported in Figure 2 could possibly represent vertical movement, or even rotations of the endosomes rather than fluctuations in the levels of Rab5. Since the endosomes are large, this could probably be addressed with non-confocal images taken at lower resolution to avoid optical slicing.

The "blinking" phenomenon of the Rab5 in cells transfected with Rab7T22N and knock-down of Rab7 is very striking. In. the cell presented in Figure 2 the Rab5 positive endosomes are in the same z-plane throughout the entire movie, we know this because the diameter of the vesicle is constant throughout the entire representative movie 2B and Figure 2. When the intensity is at its maximum the distribution of the fluorescence is uniform rather than the domain-like structure seen when the intensity is at the lowest. In fact, by analyzing these events with confocal sections is rather a strength then a disadvantage. The increased S/N ratio in confocal microscopy actually increase the quality of the measurements. The movie show that we can follow the microdomains until Rab5 redistributes/reattach to a uniform distribution. From this we conclude that the observed effect is not due to rotating endosomes.

© 2021. Published by The Company of Biologists under the terms of the Creative Commons Attribution License (https://creativecommons.org/licenses/by/4.0/).

3. I'm skeptical of statements that Rab7-positive endosomes cluster around Rab5-positive endosomes prior to maturation, simply because of the high density of both kinds of endosomes (as seen in the movies shown). A requirement for introduction of Rab7 into EE by vesicle fusion would, of course, be of major scientific interest if true. However, showing a convincing and biologically relevant effect would require some non-trivial quantitation and statistical analysis, and also demonstration of at least transient fusion of Rab7 endosomes with the EE, which I think would go beyond the scope of the current paper. The authors should be clear on the limitations of the current data, and should also cite and discuss a new paper (Langemeyer et al (2020) Elife 9:e56090) which makes an argument that, at least in an in vitro system, conversion from Rab5 to Rab7 can take place entirely through the actions of cytosolic factors and factors associated with the EE membrane.

The novel observations of the Rab7 "incoming" vesicles that seems to provide some of the necessary Rab7 to complete the maturation is a mechanism that is very interesting. We have in the revised version of the paper added a new figure (Figure 3F and G, movie 3F and G) that should shed some new light on this particular Rab7 interaction. Taken together, Figure 3E, G and F and the movies 3E, G and F, we can observe an increased Rab7 activity around the maturing endosomes. The increased activity of Rab7 positive structures, that appears like vesicles, specifically seek the maturing endosome and interact. From our analysis of these videos and many more it seems like the Rab7 recruitment appears in two phases. A first phase where the Rab7 recruited from the cytosol is distributed uniformly on the membrane and a second phase where the Rab7 is recruited through vesicular interaction, appearing in microdomains. When we carefully study these movies it seems like the maturing endosomes are "painted" in Rab7 by the vesicles recruited during the Rab5 to Rab7 transition. We think that this Rab7 activity and interaction are clearly visible in the provided figures and videos:
These events are discussed with focus on the recent findings presented in the very interesting 2020 paper by Langemeyer et al. (Langemeyer et al (2020) Elife 9:e56090).

4. The kinetic analyses in the paper follow visible (GFP-tagged) Rab5 and Rab7. This is difficult to avoid, but the authors need to make clear to the readers that they are not following the invisible pool of untagged endogenous Rab5/Rab7, and that there could be kinetic or other differences. This is particularly important with respect to the interesting observation made in the paper that Rab5 levels drop by about 50% prior to the initial arrival of tagged Rab7.

Thanks for this important comment, we are of course fully aware of that the effects we analyze here is a result of an increased level of the transfected protein. However, due to the changes induced by an increased or decreased level of the Rab proteins we find that it is likely that such an effect may be stimulated with endogenous levels of the respective proteins. We emphasize this in the discussion.

Concerning the change in the shape of the curve of the single transfected Rab5 compared to the double transfected Rab5 and Rab7, this is due to a higher expression of Rab7. When we transfected the cells with the dominant negative mutant, Rab7T22N, the level of endogenous Rab7 is also decreased. However, the remaining pool of the endogenous Rab7 is not enough for the Rab5 to Rab7 switch to occur and the Rab5 endosomes are stuck in a loop never to fully mature, hence the cyclic Rab5 level on the endosomes.
One can also notice that the drop of the intensity during the blinking event is about 50% and this significant drop cannot be accounted for by an invisible endogenous pool (see revised manuscript). Our aim is to have all these Rabs as endogenous proteins in our future work, and we will hopefully soon compare much the overexpressed GFP-fusion data with CRISPR knock in of the fusion proteins. We may then avoid protein expression higher than endogenous level and Rab-fusion proteins may work similar to the wild type molecules. There is always a catch.

5. The significance of the removal of the final pool of Rab5 in a vesicle depends in part on whether this vesicle is important to seed Rab5 into new early endosomes. Given the positive feedback loop that maintains Rab5 on the membrane, this is plausible. However, they need to acknowledge and discuss previously published papers (e.g., Cezanne (2020) Elife 9:354434) which

© 2021. Published by The Company of Biologists under the terms of the Creative Commons Attribution License (https://creativecommons.org/licenses/by/4.0/).

propose other mechanisms, e.g. ubiquitin binding, which have been proposed for recruitment of the initial Rab5.

We appreciate this comment which in fact is of help to further understand the mechanisms of our observations and this is addressed in the revised paper with proper acknowledgement.

Minor:

1) In the discussion, there is a paragraph regarding properties of endosomes during mitosis. The relevance of this needs to be clarified. Is endosomal formation/maturation frozen or otherwise modified during mitosis?
We agree with the reviewer that the relevance of this paragraph is not well explained and may also be outside the scope of this paper so therefore we have removed this from the discussion in revised version.

2) The statement in the discussion that "Both EEA1 and Rab5 are fusogenic membrane associated proteins . . ." needs modification. Both play a role in regulating membrane fusion, but, unlike SNARES and the spike proteins of enveloped viruses, are not capable of driving fusion themselves.

We agree with this comment and will address Rab5 and EEA1 as tethering proteins.

Reviewer 2 Advance Summary and Potential Significance to Field:
The manuscript of Skjeldal et al. follow in their study the behaviour of Rab5 and Rab7 during endosomal maturation. The authors use transfection with fluorophore-labeled Rab5 and Rab7 Rabs either without mutations, or with mutations favouring the GTP or GDP/empty conformation of the Rab and trace their localization relative to each other over time. As they use cells expressing the invariant chain, these cells have enlarged endosomes, facilitating their analysis of relative localization. The authors then find evidence that Rab5 detaches in domains from these enlarged endosomes before Rab7 is found on endosomes, and Rab7 thus does not seem to be required for this process. It thus seems that Rab5 positive endosomes emerge during this transition in a budding process. They also find evidence for a step-wise transition of this process.

Thanks to the reviewer for a good summary of the paper. However, we would like to clarify one misunderstanding.

The reviewer state;
"The authors then find evidence that Rab5 detaches in domains from these enlarged endosomes before Rab7 is found on endosomes, and Rab7 thus does not seem to be required for this process".
However, in the paper we state that there are two phases of the Rab5 detachment, one initial phase that are Rab7 independent and one longer, convergence phase, that is Rab7 dependent. If Rab7 is not present on the endosomes they will not go through the Rab5-Rab7 switch and result in the Rab5 oscillations shown in Figure 2.

Reviewer 2 Comments for the Author:
The authors rely in their study primarily on GFP or RFP-tagged Rab5 and Rab7, which are apparently overexpressed in cells. Their fluorescence microscopy looks well done and controlled and indeed shows a striking transition from endosome-associated Rab5 to Rab5 domains and Rab5-positive and Rab7 negative vesicles. This apparent vesicular exchange is quite different from the model, in which Rab7 recruitment and Rab5 release are coupled (Rink et al., 2005, Poterayev et al, 2010). My problem with the study is rather that I am not sure what I really learn from this. In all cells, the wild-type protein is still present. If the authors really want to make a point in the vesicle exchange of Rab5 and Rab7, then they need to interfere with the endogenous protein by interfering with the Rab7 GEF or the Rab5 GAP, or by inactivating the endogenous Rab5 and expressing an siRNA-resistant Rab5 instead. Of course, tracing Rab5 and Rab7 has a value of its own as the extra copies of each protein may reflect the movement of endosomal membranes, yet the authors do not trace membrane per se in their assays, but the fluorophore-labelled structures and apparently do strong overexpression. At this point, this is an interesting observation, where I

© 2021. Published by The Company of Biologists under the terms of the Creative Commons Attribution License (https://creativecommons.org/licenses/by/4.0/).

miss a lot of controls. I will list some of my concerns that may help them to frame the study better.

1. The study starts out with a misunderstanding of the Rab7 GEF, which is a bit unfortunate. Several studies showed that the Mon1-Ccz1 complex is the Rab7 GEF in yeast, Drosophila, plants and humans, a crystal structure is available of Mon1-Ccz1 and Rab7, and a recent study showed clearly that Rab5 is required for Rab7 activation (Langemeyer et al., 2020). Also Nordmann et al., 2010 showed already that Vps39 has no GEF activity. This needs to be corrected in their introduction and discussion part.

We agree that this is unfortunate (and a bit embarrassing) and we are glad that the reviewer commented on this. We have corrected this in the revised version of the paper.

2. The authors describe a two-phase release process (Figure 1C). They should repeat the experiment in cells, where they silenced the Mon1-Ccz1 GEF complex and follow Rab7 in parallel. I expect that the Rab7 is then primarily in the cytosol, which would be a good control for their assays.

We agree with the reviewer that this experiment could have been interesting and was also in our mind during the project. The reason why we think this is not necessary is that it will be a similar to the use of Rab7T22N. When the dominant negative Rab7 is expressed the Rab7T22N is only in the cytosol and the maturation of the endosomes are inhibited and the size of the Rab5 positive endosomes increase. If the endogenous level of Rab7 was high we would be able to observe endosomes that mature, but they do not. Instead, as show in the figure 2 the Rab5 detaches and reattaches in a cyclic manner. As a supporting experiment we did a knock down of Rab7, which reproduced the Rab5 off/on cycle. In our opinion, to perform further controls would not increase significantly the conclusion in of this part of our study.

3. Along this line, the authors mention that Rab7 is not required for Rab5 release.
Both yes and no, Rab7 is absolutely necessary for the convergence phase of Rab5 into microdomains and finally the transfer of Rab5 to the to the new endosome. If Rab7 is RNAi silenced or endosomal binding is inhibited by dominant negative Rab7 the maturation will not occur. The initial diffusion like detachment of Rab5 is on the other hand not Rab7 dependent. This is corroborated by the data showing that Rab5 is started to detach and reattach again when active Rab7 is absent. The mCh-Rab5 seem to start the convergence phase and establish Rab5 domains but due to the lack of Rab7 the convergence phase is not completed, and the mCh-Rab5 reattaches to the membrane. There seem to be a sequence of events that starts but are not completed, but we have not identified the potential different interactors, except that lack of active Rab7 causes the process to stop, and restart in a cyclic manner.

However, they still have the wild-type protein in the background, and just following the overexpressed Rab7 does not really make this point.

When we carried out this experiment we were also thinking the same direction as the reviewer. However, when blocking the endogenous Rab7 present in the cells after transfection with **dominant negative** Rab7T22N or the KD of Rab7 any remaining endogenous active Rab7 is not sufficient for the endosomes to complete the Rab5 to Rab7 transition. Specifically shown in figure 2. There is still an endogenous Rab7 level but it doesn't seem to be enough to maintain a regular Rab5 to Rab7 transition.

4. Figure 1 – graphs in A-D. The authors label only the Y-axis with the mCherry Rab5, but also trace Rab7 (GFP-tagged), and this has to be added as an information.

Thank for pointing this out. This is corrected in the new version.

5. Figure 3D is missing, and I can thus comment only on the text. In fact, I am not sure what to learn from this. Does this mean that EGFR is recycled once taken up into the cell? I always thought that EGFR moves directly to the MVB and the lysosome?

© 2021. Published by The Company of Biologists under the terms of the Creative Commons Attribution License (https://creativecommons.org/licenses/by/4.0/).

We apologize for this, the figure got lost when we were asked by the editorial staff to remake the figures into fewer pages. As seen in the revised version, Fig. 3D illustrates that the newly formed endosome is a fully functional early endosome that will acquire EGF for endosomal sorting.

6. In Figure 3C and F, the authors trace EEA1. As EEA1 binds Rab5-GTP, this is one of the few cases, where an apparent cargo and thus membrane is followed. Based on this, the Rab5-positive structures contain active Rab5, indicating indeed that Rab5 is not inactivated when Rab7 arrives. Previous work indicates that Rab5 and Rab7 bind also retromer. It would be of interest if the authors repeat their analysis when they inactivate retromer as the separation of endosomal domains likely requires this complex.

This is an interesting model and will be included in future work to elucidate the full molecular events of this crucial stage of endosomal maturation, but outside the scope of this first paper reporting the “recycling” of active Rab5, as we show in our FRAP experiments.

7. I find it important that the authors trace the identity of their membranes with additional markers beyond Rab5 and Rab7 such as the FYVE domain to get additional information on its identity.

We agree that this is essential and we have added five figures of supplementary data with additional early endosomal associated proteins. To be more precise, we provide new supplementary figures with Rab22, Rab4, Hrs and Eps15 that follow a similar mechanism as discussed above in response to reviewer 1.

The convergence maturation and the transfer of early endosomal associated protein to induce the formation of an early endosome seems to be a general mechanism to transfer the necessary membrane proteins, to a fully functional early endosome.

Second decision letter

MS ID#: JOCES/2020/254185

MS TITLE: De novo formation of early endosomes during Rab5 to Rab7 transition

AUTHORS: Frode M Skjeldal, Linda Haugen, Duarte Mateus, Dominik Michael Frei, Anna Roedseth, Xian Hu, and Oddmund Bakke
ARTICLE TYPE: Research Article

We have now reached a decision on the above manuscript.

To see the reviewers' reports and a copy of this decision letter, please go to: https://submit-jcs.biologists.org and click on the 'Manuscripts with Decisions' queue in the Author Area. (Corresponding author only has access to reviews.)

As you will see, the reviewers gave favourable reports but one of the reviewers suggested that you add an additional sentence or two in your discussion on how your proposed model differs from that suggested by Cezanne (2020). I hope that you will be able to carry these out, because I would like to be able to accept your paper.

We are aware that you may be experiencing disruption to the normal running of your lab that makes experimental revisions challenging. If it would be helpful, we encourage you to contact us to discuss your revision in greater detail. Please send us a point-by-point response indicating where you are able to address concerns raised (either experimentally or by changes to the text) and where you will not be able to do so within the normal timeframe of a revision. We will then provide further guidance. Please also note that we are happy to extend revision timeframes as necessary.

© 2021. Published by The Company of Biologists under the terms of the Creative Commons Attribution License (https://creativecommons.org/licenses/by/4.0/).

Please ensure that you clearly highlight all changes made in the revised manuscript. Please avoid using 'Tracked changes' in Word files as these are lost in PDF conversion.

I should be grateful if you would also provide a point-by-point response detailing how you have dealt with the points raised by the reviewers in the 'Response to Reviewers' box. Please attend to all of the reviewers' comments. If you do not agree with any of their criticisms or suggestions please explain clearly why this is so.

Reviewer 1

Advance summary and potential significance to field

This paper suggests that the early-to-late endosome transition (from Rab5+ to Rab7+) is facilitated by direct delivery of Rab7 from Rab7+ endosomes to Rab5+ endosomes, in contrast to the standard model for maturation where Rab7 is recruited directly from cytosol. In my view, there are technical limitations to the study which reduce the strength of the conclusions that can be drawn, and the paper will likely be controversial. However, the current manuscript is greatly improved, and the experimental designs have been strengthened compared to the previous manuscript. I believe this is worthy of follow-up by the scientific community, as the model proposed is logical given the known existence of positive feedback loops maintaining these Rabs on endosomal membranes, and the scientific questions that are not definitively answered at this point will be good starting points for future research.

Comments for the author

One minor comment.

Cezanne (2020) is now cited as I suggested, but there should be at least an additional sentence or two of discussion of how the model proposed in that paper differs from that suggested in the current ms.

Reviewer 2

Advance summary and potential significance to field

see my previous comments

Comments for the author

The authors revised the manuscript according to the suggestions I provided. I have no further comments and recommend publication.

Second revision

Author response to reviewers' comments

We have added a few sentences to the discussion as requested. See revised manuscript.

Third decision letter

MS ID#: JOCES/2020/254185

MS TITLE: De novo formation of early endosomes during Rab5 to Rab7 transition

© 2021. Published by The Company of Biologists under the terms of the Creative Commons Attribution License (https://creativecommons.org/licenses/by/4.0/).

AUTHORS: Frode M Skjeldal, Linda Haugen, Duarte Mateus, Dominik Michael Frei, Anna Roedseth, Xian Hu, and Oddmund Bakke
ARTICLE TYPE: Research Article

I am happy to tell you that your manuscript has been accepted for publication in Journal of Cell Science, pending standard ethics checks.

© 2021. Published by The Company of Biologists under the terms of the Creative Commons Attribution License (https://creativecommons.org/licenses/by/4.0/).
